# Hafnium-Doped Mesoporous Silica as Efficient Lewis Acidic Catalyst for Friedel–Crafts Alkylation Reactions

**DOI:** 10.3390/nano9081128

**Published:** 2019-08-05

**Authors:** Yao-Bing Huang, Yu-Jia Luo, Fei Wang

**Affiliations:** 1 Jiangsu Co-Innovation Center of Efficient Processing and Utilization of Forest Resources, Nanjing Forestry University, Nanjing 210037, China; 2 College of Chemical Engineering, Nanjing Forestry University, Nanjing 210037, China; 3 Department of Chemical and Biomolecular Engineering, University of California-Berkeley, Berkeley, CA 94706, USA

**Keywords:** Friedel–Crafts alkylation, hafnium-doped mesoporous silica, benzyl alcohol, aromatics, Lewis acid

## Abstract

The development of an efficient solid catalyst for Friedel–Crafts (FC) reactions is of great importance to organic synthetic chemistry. Herein, we reported the hafnium-doped mesoporous silica catalyst Hf/SBA-15 and its first use for Friedel–Crafts alkylation reactions. Catalysts with different Si/Hf ratios were prepared and characterized, among which Hf/SBA-15(20) (Si/Hf = 20:1) was the most active catalyst, offering up to 99.1% benzylated product under mild reaction conditions. The influences of reaction conditions on the product were systematically investigated and compared. Pyridine-IR characterization of the catalyst showed that Lewis acid formed the primary active sites for the Friedel–Crafts alkylation reaction. X-ray photoelectron spectroscopy (XPS) characterization revealed that the electron shift from the Hf center to the silica framework resulted in a more active Lewis metal center for FC reactions. Moreover, the catalyst was successfully applied to the alkylation reaction with different alcohols and aromatic compounds. Finally, the Hf/SBA-15(20) catalyst also showed good recyclability in the recycling runs, demonstrating its high potential of being used for large scale FC reactions in the industry.

## 1. Introduction

Friedel–Crafts (FC) alkylation is one of the most important organic chemistry reactions that allow the production of substituted aromatic compounds through the formation of new C–C bonds [1,2]. Many FC alkylated aromatic products, such as diphenylmethane and derivatives synthesized by the FC alkylation of benzene and various aromatic substrates, are used as key intermediates for the production of pharmaceutical and chemical compounds in the industry [3]. In addition, the FC reaction can also be applied to the synthesis of renewable biofuels from natural biomass resources, which yields higher carbon fuel precursors (e.g., C15) from small platform molecules (e.g., C5) that fall within the carbon range of diesel and jet fuels [4].

Typically, the FC alkylation reaction is catalyzed by homogeneous acid catalysts (e.g., H_2_SO_4_, HCl, HF, AlCl_3_, FeCl_3_) [5], which also inherit the disadvantages of using traditional liquid acid catalysts, such as being highly corrosive, non-recyclable, and economically unfriendly. That aside, alkyl halides are also frequently used alkylated reagents that may form HX acids after the reaction, resulting in more cost-inefficient post-run treatments to minimize its impact on the environment [6]. To overcome these disadvantages, there are increasing interests of using heterogeneous acids as catalysts, together with the use of alcohol [7] or ester [8] as alkylating reagent, which make the FC reaction more environmentally benign and cost-efficient. During the past few years, several types of Bronsted- and Lewis-type solid catalysts have been identified with good reactivities in FC alkylation reaction, including supported heteropolyacid (HPA) [9], supported metal salts [10], zeolites [11], and mesoporous solid acid catalysts [12]. For example, Lingaiah et al. [13] reported tin-exchanged heteropoly tungstate was successfully applied to the benzylation of arenes with benzyl alcohol and almost full conversion and quantitative products were obtained at 393 K of reaction temperature. Sugi et al. [14] reported the rare earth metal triflates (e.g., Sc(OTf)_3_, Hf(OTf)_3_) supported on MCM-41 for FC reactions and over a 90% yield of target products could be obtained at 353 K. Kaper et al. [15] reported the use of mixed oxide SiO_2_-ZrO_2_ for the FC alkylation of anisole and benzylic or allylic alcohols and they claimed the importance of creating a mesoporous structure of the catalysts, which enabled better mass diffusion during the reaction. Of all these solid catalysts, mesoporous Lewis type catalysts are more promising and practical for FC reaction in terms of their high reaction efficiency, better thermal stability, and recyclability.

During our previous work on the alkylation of 2-methylfuran with aldehyde to form a diesel precursor, the Zr-doped SBA-15 catalyst synthesized by sol–gel methods possessed good Lewis acidity and exhibited outstanding reactivity in the alkylation reaction [16]. To further explore the potential of mesoporous materials supporting Group IVB metals in the alkylation reaction, we extended our research by using Zr and Hf as the center metals for the FC reaction with benzyl alcohols as the benzylating agents. To our delight, an almost quantitative yield of the alkylated products was achieved with the Hf/SBA-15 catalyst. To the best of our knowledge, this was the first time that a highly ordered mesoporous Hf catalyst was used for this reaction. To gain further insight into the reaction, we herein reported the use of a sol–gel method to prepare Hf/SBA-15 catalysts for the FC alkylation reaction of benzyl alcohol and aromatics to produce diphenylmethane derivatives. Several catalysts with different Si/Hf ratios were prepared, characterized, and compared in the alkylation reaction. The influences of reaction parameters on the reaction efficiency were also evaluated. Finally, catalyst recycling and characterization were also included.

## 2. Materials and Methods

### 2.1. Materials

Poly(ethylene glycol)-block-poly(propylene glycol)-blockpoly(ethylene glycol) (Pluronic P123, Sigma–Aldrich Inc., Shanghai, China), tetraethylorthosilicate (TEOS, TCI Inc., Shanghai, China), and HfCl_4_ (Sigma–Aldrich Inc., Shanghai, China) were used. All other common reagents and solvents were bought from a chemical company (Aladdin Inc., Shanghai, China).

### 2.2. Catalysts Characterization

Nitrogen adsorption–desorption isotherms were performed on the Autosorb-iQ2 from Quantachrome company. Catalysts were degassed at 300 °C under nitrogen before absorption. Small XRD spectra were recorded on an X’pert (PANalytical) diffractometer at 40 kV and 200 mV, over a 2θ range from 0.5 to 5°, using a step size of 0.004°. Wide-angle resolution patterns were obtained over 10–80°, using a step size of 0.02°. XPS spectra were recorded on a Kratos Axis Ultra DLD photoelectron spectrometer. The acid types of catalysts were determined by Pyridine-adsorbed Fourier Transform Infrared Spectroscopy (Py-FTIR), using a Bruker Tensor 27 spectrometer. Transmission electron microscopy (TEM) characterization was conducted on a JEOL JEM-1400 UHR microscope. Scanning electron microscope (SEM) characterization was conducted on a JEOL JSM-7600F. Thermogravimetric analysis (TGA) was conducted on the Shimadzu DTG-60A thermal analyzer.

### 2.3. Catalysts Preparation

Hf/SBA-15 catalysts were synthesized using a sol–gel method as reported in [17]. Typically, 2 g P123 was dissolved in 75 mL hydrochloric acid (1.6 mol/L) and stirred for 3 h in a 40 °C water bath. After that, 4.25 g TEOS was then added with a certain amount of Hf precursor. The resulting mixture was stirred for 24 h and then subjected to hydrothermal aging at 100 °C for 24 h. After cooling down to room temperature, the resulted solids were filtered, dried in the oven, and finally calcined at 550 °C for 6 h. Catalysts were denoted as Hf/SBA-15(X), where X refers to the mole ratio of Si/Hf.

HfO_2_ was prepared according to the reported procedure in [18]. Typically, ammonium hydroxide was dropwise added into the aqueous solution of HfCl_4_ (2 mmol in 10 mL deionized water) until pH reached 8–9, and continued to be stirred at room temperature for 2 h, before being subjected to hydrothermal treatment at 120 °C for 6 h. After that, the solids were filtered and washed with deionized water and dried overnight at 80 °C. Finally, the catalyst was calcined at 550 °C for 4 h.

Hafnium phosphate catalyst was prepared according to the following procedure: Firstly, KH_2_PO_4_ solution (4 mmol in 10 mL deionized water) was dropwise added into the HfCl_4_ aqueous solution (2 mmol in 10 mL deionized water), stirred at room temperature for 2 h, and then subjected to hydrothermal treatment at 120 °C for 6 h. The resulting solids were filtered, washed with deionized water, and dried at 80 °C overnight. Finally, the catalyst was obtained after the calcination of the solids at 550 °C for 4 h.

### 2.4. Catalytic Reactions

In a typical reaction, 80 mg Hf/SBA-15(20) catalyst, 0.5 mmol benzyl alcohol, and 3 mL toluene were charged into a 35 mL reaction tube and stirred at 120 °C for 6 h. After the reaction was complete, the reaction mixture was sampled and analyzed by gas chromatography (Agilent, 7890A), equipped with a DB-5 capillary column (30 m × 0.25 mm × 0.25 μm, Agilent) and a flame-ionization detector (FID). Naphthalene was used as the internal standard to calculate the reaction conversions and product yields.

## 3. Results

### 3.1. Catalysts Characterization

**Figure 1:**
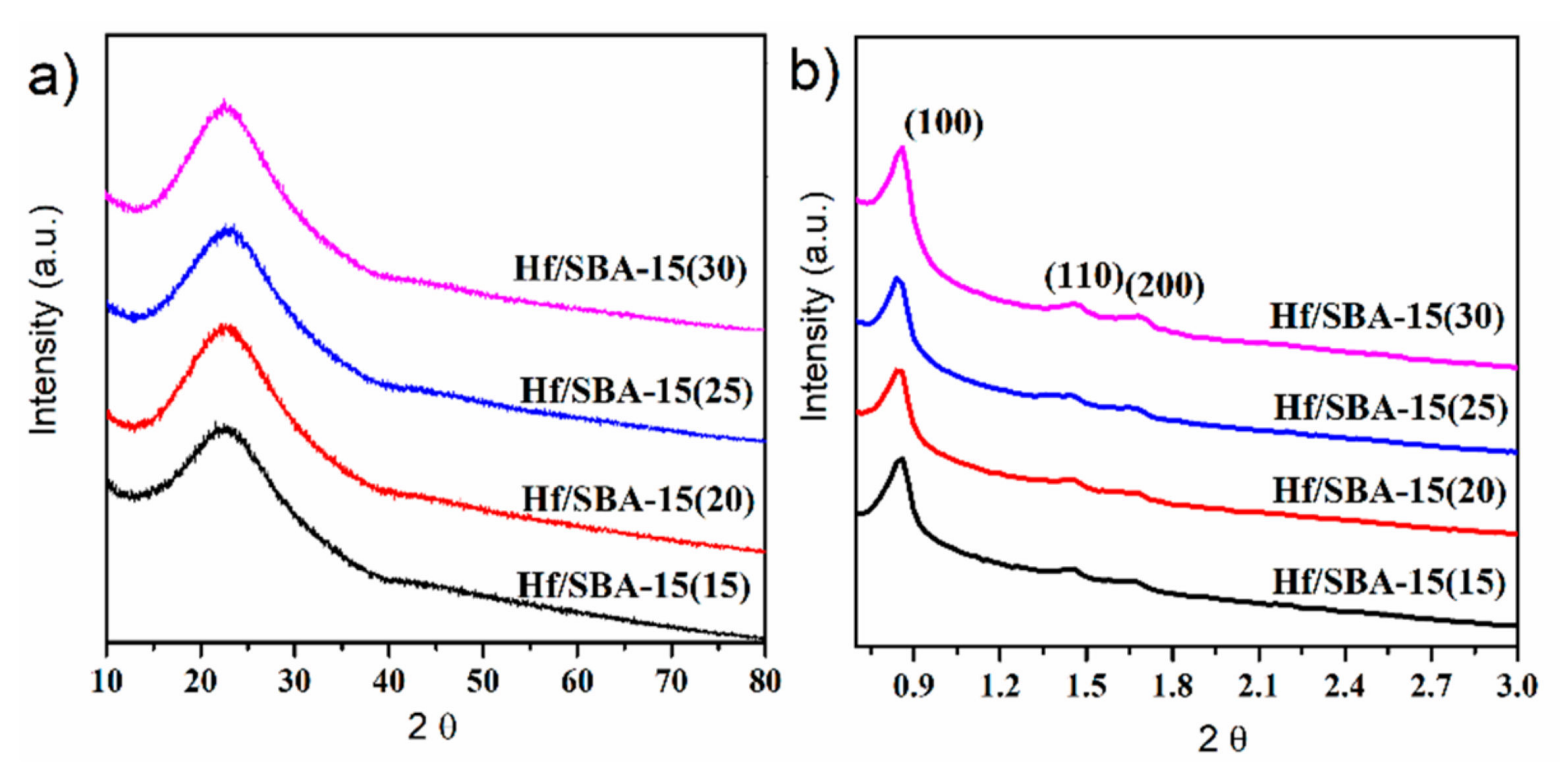
(**a**) Wide and (**b**) small angle XRD characterization of Hf/SBA-15 catalysts.

Firstly, the as-prepared Hf/SBA-15(X) catalysts were subjected to XRD characterization, as presented in Figure 1. Wide diffraction peaks around 20–30° were observed across all Hf/SBA-15 catalysts with different Si/Zr ratios, which indicated the amorphous structure of the obtained catalysts [19]. No typical peaks corresponding to metal oxide (i.e., HfO_2_) were found, further suggesting the homogeneous dispersion of Hf metal within the catalysts. In the small angle XRD spectra, all Hf/SBA-15 catalysts showed three diffraction peaks around 0.9°, 1.6°, and 1.8°, which can be assigned to the (100), (110), and (200) diffraction peaks of a two-dimensional (2D) hexagonal p6mm structure, respectively. They were also the characteristic patterns of SBA-15, furthering confirming the successful construction of the SBA-15 framework [20]. In addition, the orderliness of the SBA-15 structure was well reserved after the incorporation of Hf metal, which might benefit the mass diffusion within the catalyst’s structure during the reaction.

The textural properties of the as-prepared Hf/SBA-15 catalysts were presented in Table 1. The catalysts showed decreased Brunauer–Emmett–Teller (BET) surface area from 811.3 to 624.4 m^2^/g, as well as the pore volume from 1.4 to 1.1 cm^3^/g, along with the increase of Hf content in the catalyst (Si/Hf ratio decreased from 30 to 15). These results were predictable as more Hf metal would replace Si to form an SBA-15 framework, which had a different atom size or coordination mode as compared to Si that would alter the native SBA-15 structure. Furthermore, some of the small Hf oxide aggregates may be formed and block the pores, due to the introduction of more Hf metal. Aside from that, all the catalysts still possessed high BET surface areas over 600 m^2^/g, which would be beneficial for the alkylation reaction (Table 1).

**Figure 2:**
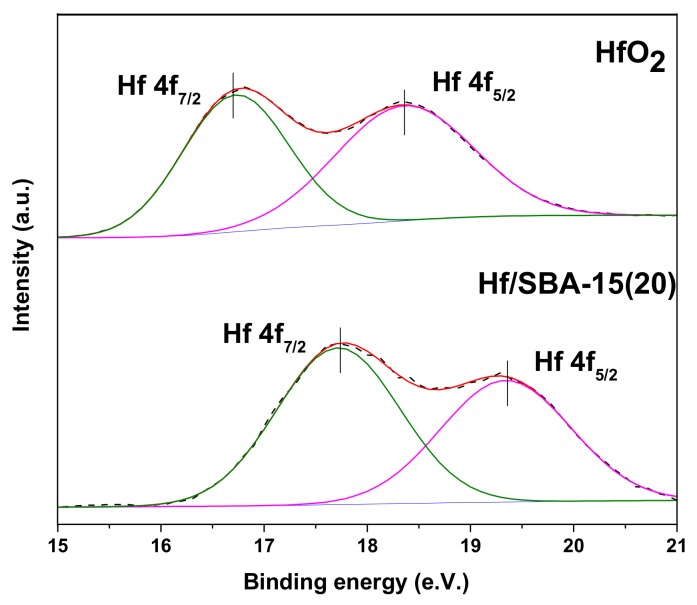
XPS spectra of Hf in HfO_2_ and Hf/SBA-15(20) catalyst.

XPS characterization of the representative catalyst Hf/SBA-15(20) was investigated, together with the metal oxide HfO_2_ as a control sample, to provide detailed insights into the chemical states of the Hf within the catalysts. As shown in Figure 2, the binding energy value around 16–22 eV was the Hf 4f peak of the Hf/SBA-15(20) catalyst, which can be further deconvoluted into Hf 4f_7/2_ and Hf 4f_5/2_, with the binding energies of 17.7 and 19.3 eV, respectively. For HfO_2_, these two major binding energy values were located at 16.8 and 18.4 eV, respectively. By comparing the binding energies of Hf species in these two different catalysts, clear shifts of the binding energies to higher values were observed from HfO_2_ to Hf/SBA-15, which was indicative of more electron deficiency of Hf metal in the Hf/SBA-15 catalyst. This was caused by the electron transfer from Hf to silica, due to the strong higher electronegativity of silicon [21], thereby leading to a more positively charged Hf metal center. This would also promote the Lewis acidity of the Hf center and increase the catalyst’s reactivity toward the FC reaction.

**Figure 3:**
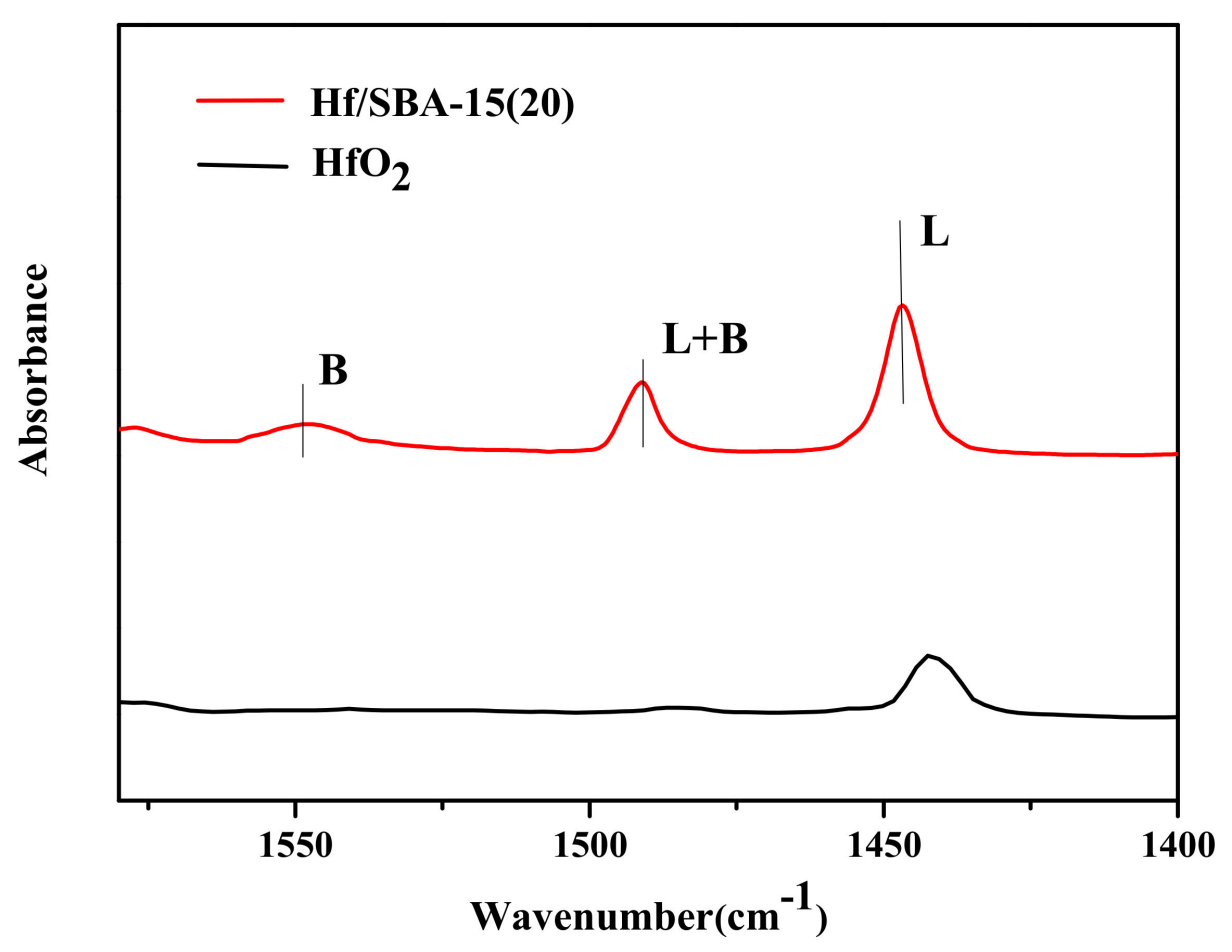
Pyridine-IR of Hf-SBA/15(20) and HfO_2_ catalysts.

The acid properties of Hf/SBA-15(20) and HfO_2_ were investigated by Py-IR characterizations, as revealed in Figure 3. For the Hf/SBA-15(20) catalyst, three peaks corresponding to different types of acids were observed. The peak at 1450 cm^−1^ originated from the Lewis acidic sites, whereas the wide peak at 1540 cm^−1^ represented the Brønsted acidity of the catalyst. The peak at 1490 cm^−1^ was assigned to the mixed Brønsted and Lewis acidic sites. According to the quantitative analysis of the desorbed pyridine, the amount of Lewis acid sites of Hf/SBA-15(20) was calculated to be 176.3 µmol/g and the amount of Brønsted acid sites was 60.7 µmol/g, indicating the dominated Lewis acid types of the Hf/SBA-15(20) catalyst. For non-mesoporous HfO_2_, only weak Lewis acidic sites were detected on the catalyst’s surface and the amount of acid sites was 11.4 µmol/g, much lower than that of mesoporous Hf/SBA-15(20). These results clearly showed that the immobilization of metal sites into the framework of mesoporous material can expose more metal sites, thereby increasing the total acid sites on the catalyst’s surface. Moreover, by using a silica framework, the electron transfer from Hf metal to silica, as indicated in the XPS result, also promoted the Lewis acidity of the Hf center, making Lewis acid sites the dominant acid type of the catalyst, which would also benefit the alkylation reaction. These characterizations further highlighted the importance of mesoporous silica frameworks for the preparation of highly efficient metal catalysts.

**Figure 4:**
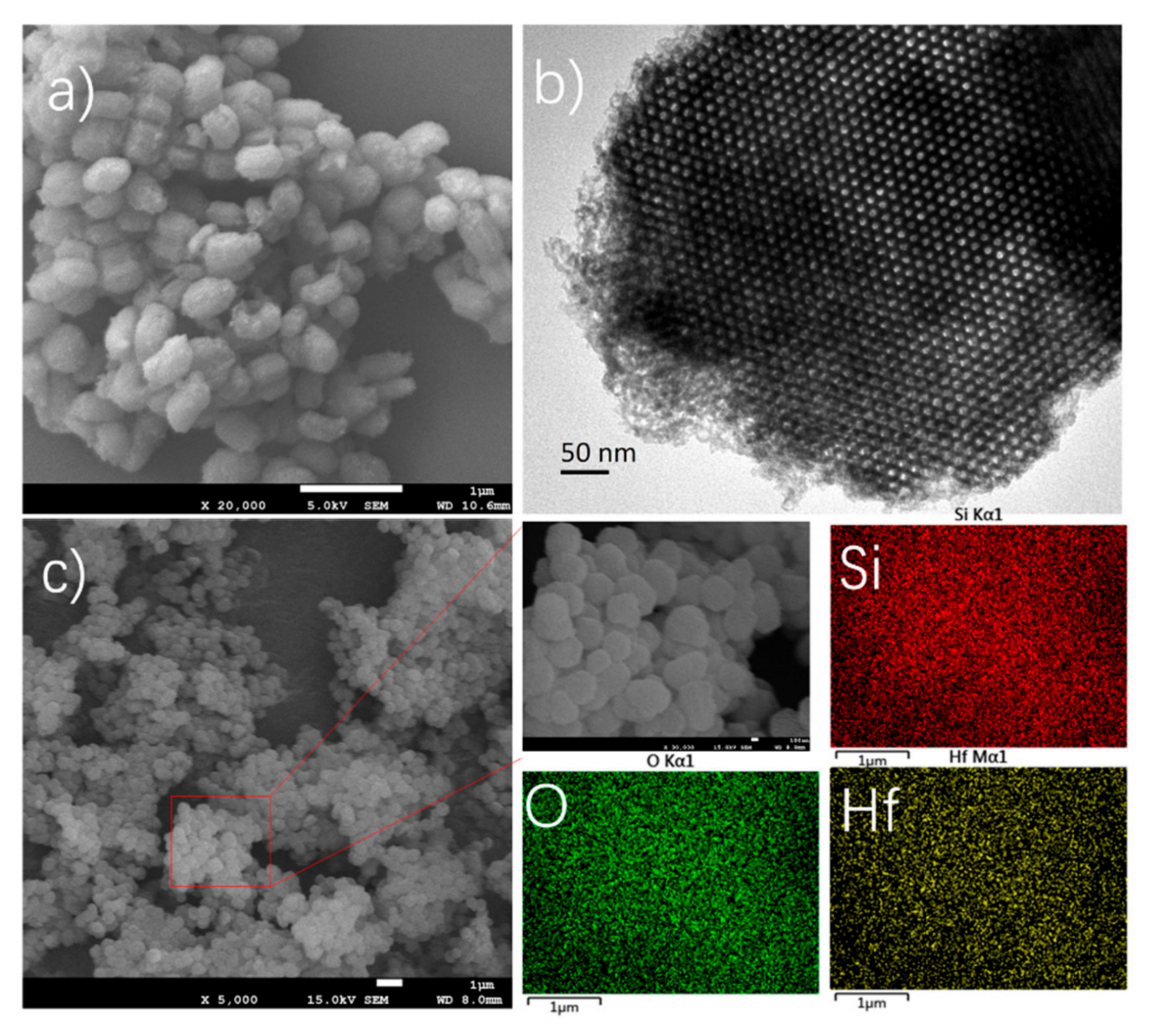
Mesoporous structure of the representative Hf/SBA-15(20) catalyst. (**a**) SEM; (**b**) TEM; (**c**) elemental mapping.

SEM and TEM characterizations of typical catalyst Hf/SBA-15 (20) were also provided to elucidate structure characteristics in Figure 4. The catalyst had a rod shape with a mean size of 600 nm in length (Figure 4a). TEM spectra showed 2D pore distribution in sight, along with darker areas within the framework of the catalyst (Figure 4b). These results indicated that the Hf metal were homogeneously dispersed in the catalysts, without forming visible metal oxide. The catalyst was also subjected to the STEM-mapping characterization, and well-dispersed metal sites were observed, indicating the homogeneous immobilization of the metal within the structure of the silica framework.

### 3.2. Catalyst Screening

**Scheme 1:**
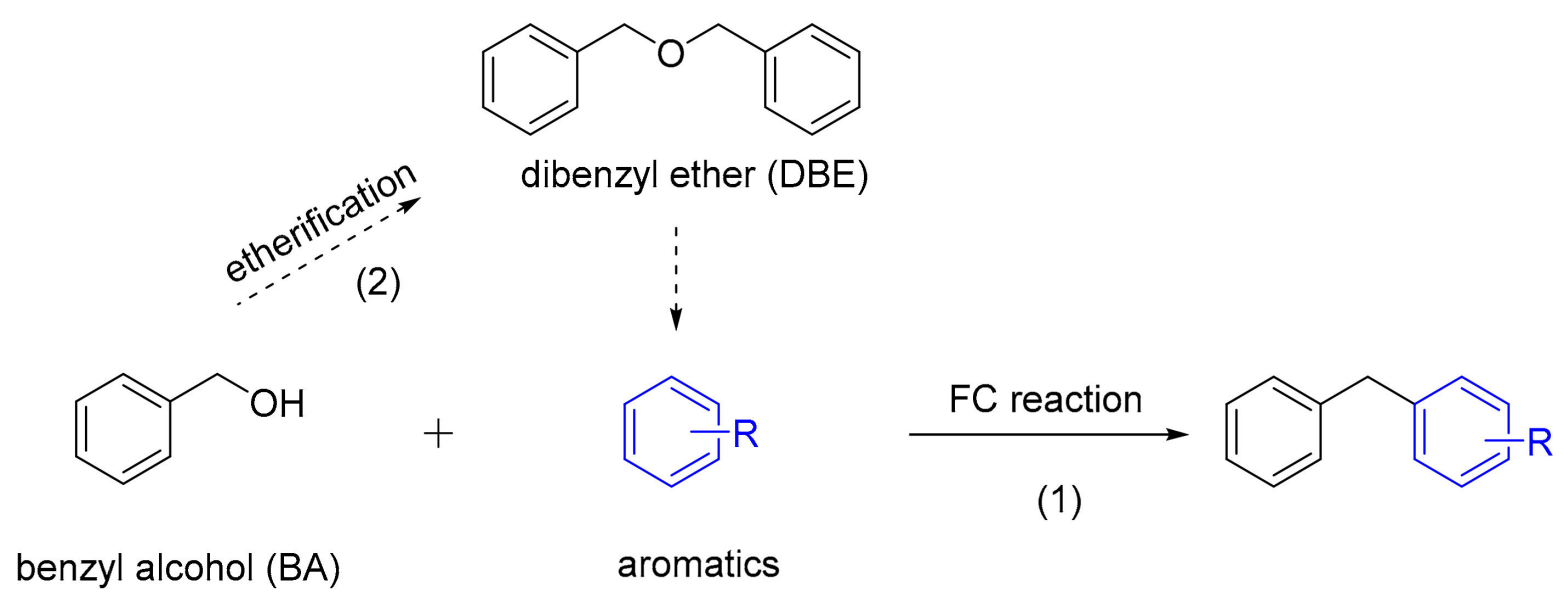
The Friedel–Crafts (FC) alkylation reactions of benzyl alcohol and toluene.

Firstly, benzyl alcohol and toluene were selected as the model compound for the FC alkylation reaction over a series of solid acid catalysts. The main reaction steps involved in the reaction were presented in Scheme 1. Table 2 showed the catalytic performances of different solid acid catalysts for FC reaction under neat reaction conditions at a fixed metal loading. The reaction conversion and BA yield were analyzed by GC and 1H-NMR (SI). For the blank test, pure SBA-15 material was subjected to the FC reaction, and no product was formed, indicating the necessity of employing metal sites for the reaction (Table 2, Entry 1). When we subjected our previously used Zr/SBA-15 catalyst, a Lewis acidic catalyst that was successfully applied to the alkylation of 2-methylfuran with furfural to produce a C15 diesel precursor, to the FC reaction, an 89.3% yield of the target product was obtained (Table 2, Entry 2). This result encouraged us to extend our research by exploring more metal-doped mesoporous catalysts for FC reactions. For W/SBA-15, an 83.5% yield of the target products could also be obtained (Table 2, Entry 3). However, Fe/SBA-15 showed no catalytic reactivity. When we turned to the Hf/SBA-15 catalyst, product yields over 90% could be obtained across all the catalysts with different ratios (Table 2, Entries 5–8). Particularly, when the Si/Hf ratio was 20, the catalyst exhibited the highest reactivity, offering an almost quantitative product yield (99.1%). By comparing the structure characteristics with other Hf/SBA-15 catalysts with different Si/Hf ratios, Hf/SBA-15(20) seemed to possess an approximate BET surface area or pore volume/diameter ratio that enabled better catalytic performance of Hf sites. According to the above Py-IR characterization, Lewis acidity of Hf/SBA-15 should be primarily responsible for the FC reaction, which activated the alcohol to generate a positively charged carbon center to undergo an electrophilic substitution reaction on aromatic rings. By comparing our results with the previously reported FC benzylation reactions with Lewis type catalysts, such as SiO_2_-ZrO_2_ and Al/SBA-15, Hf/SBA-15 possessed higher reactivity under relatively mild reaction conditions (Table S1). This catalyst was further applied to the condensation of furfural and 2-methylfuran where a 96.6% product yield was achieved, higher than that with the Zr/SBA-15 catalyst (Scheme S1), which further confirmed the higher reactivity of Hf over Zr when immobilized in the framework of SBA-15 supports [16].

Additionally, various Hf catalysts (i.e., HfCl_4_, HO_2_, HfO(PO_4_)_2_, and HfO(OH)_2_) were also tested for the FC reaction (Table 2, Entries 9–12). However, all these catalysts showed almost no reactivity toward the FC reaction. These results were probably caused by the lower Lewis acid density or lower pore structure of the catalyst that was unable to activate benzyl alcohol for the reaction, which, on the other hand, emphasized the necessity of creating a mesoporous silica framework for promoting the Lewis acidity of Hf sites. Some commercially available acidic catalysts were also compared with our Hf/SBA-15(20). We found that only a moderate yield could be achieved over Amberlyst-15 and H-β, with even less yield achieved over H-ZSM-5, implying that the catalytic efficiency of Hf/SBA-15(20) in the FC alkylation of benzyl alcohol and toluene was very high.

The influence of substrate ratio was also evaluated by reducing toluene usage from 3 to 1.5 or 1 mL (Table S6). A gradual decrease in the product yields was observed along with the decrease of toluene usage, which was common in the FC alkylation reaction. Fortunately, by further prolonging the reaction time, a satisfying product yield could be achieved even with the lower toluene usage. In terms of the recyclability of the aromatics and economics of the process, an excessive loading of aromatics was acceptable.

### 3.3. Reaction Pathway

**Figure 5:**
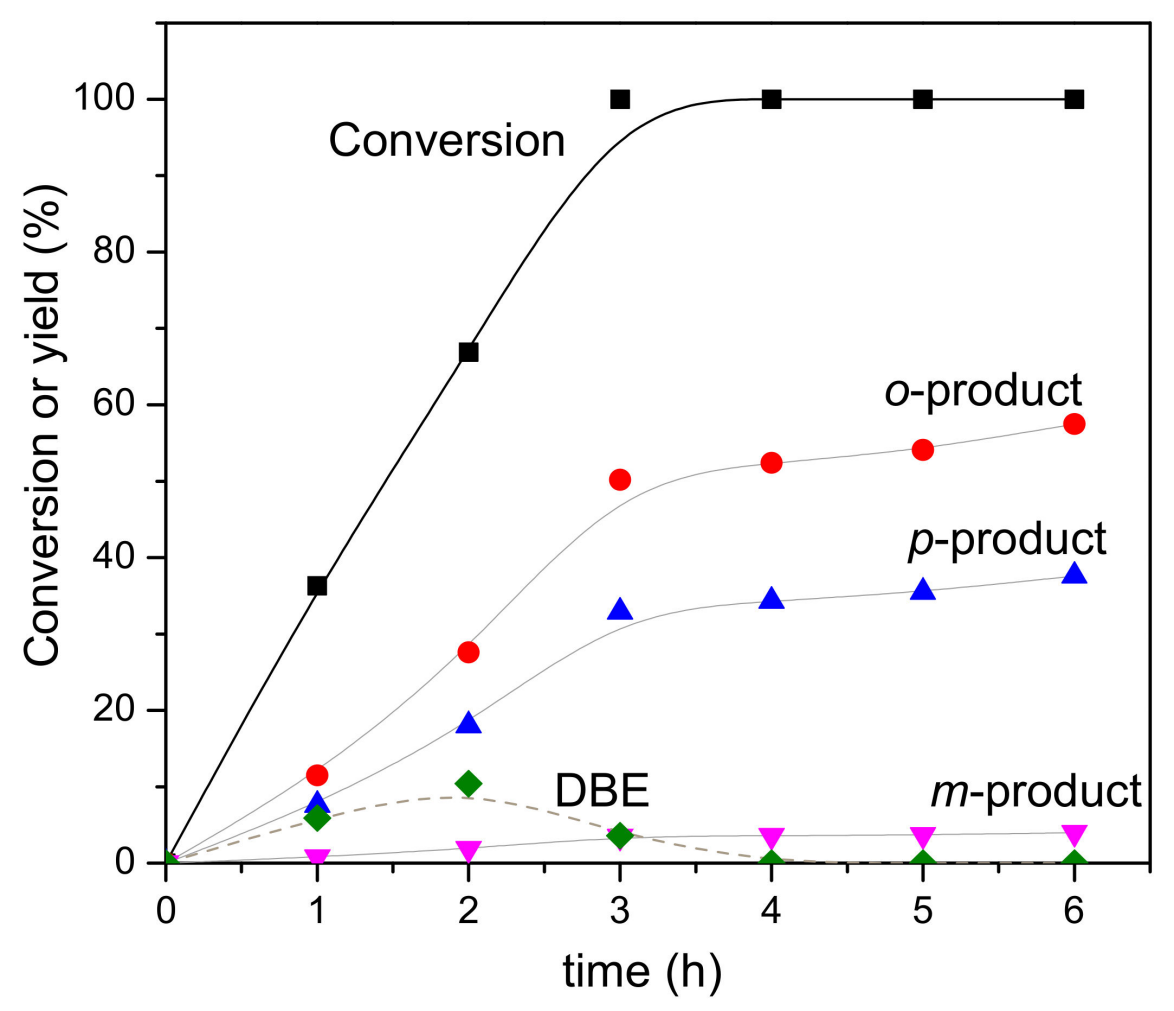
Reaction evolution over Hf/SBA-15(20) catalyst. Conditions: 0.5 mmol benzyl alcohol, 3 mL toluene, 120 °C, 80 mg Hf/SBA-15(20).

The reaction pathway was investigated by monitoring the reaction over time. Figure 5 showed the product evolution in the reaction over the Hf/SBA-15(20) catalyst. After a short reaction time of 1 h, the benzylated products could be detected with a total yield of 20%, accompanied by the side product, dibenzyl ether (DBE). This side product was generated by self-condensation of benzyl alcohol over acidic sites at elevated temperature, which is common in an FC reaction [22]. Luckily, this side product could also act as the electrophile to undergo FC reaction by releasing a PhCH_2_+ to attack the aromatic ring of toluene. As the reaction proceeded, the yield of DBE gradually decreased, while that of the target benzylated products continued to increase, reaching the highest yield of 99.1% at 6 h. These results clearly suggested that the etherification of benzyl alcohol and the direct FC reaction occurred in parallel, in the presence of the Hf/SBA-15 catalyst. The etherification product DBE was further activated by Hf sites and contributed to the alkylation reaction, as elucidated in route 2. To further asses the difference between Zr/SBA-15 and Hf/SBA-15 in catalyzing this reaction, we also traced the FC reaction by using a Zr/SBA-15 catalyst, which exhibited similar product evolution trends, but with slower reaction rates, offering 89.3% product yields within 6 h reaction time (Figure S1). This was probably due to the stronger acidity of Hf/SBA-15 over Zr/SBA-15, as evidenced by the Pyridine-IR characterization (Figure S2) that promoted the whole reaction rates.

### 3.4. Effect of Catalyst Loading

**Figure 6:**
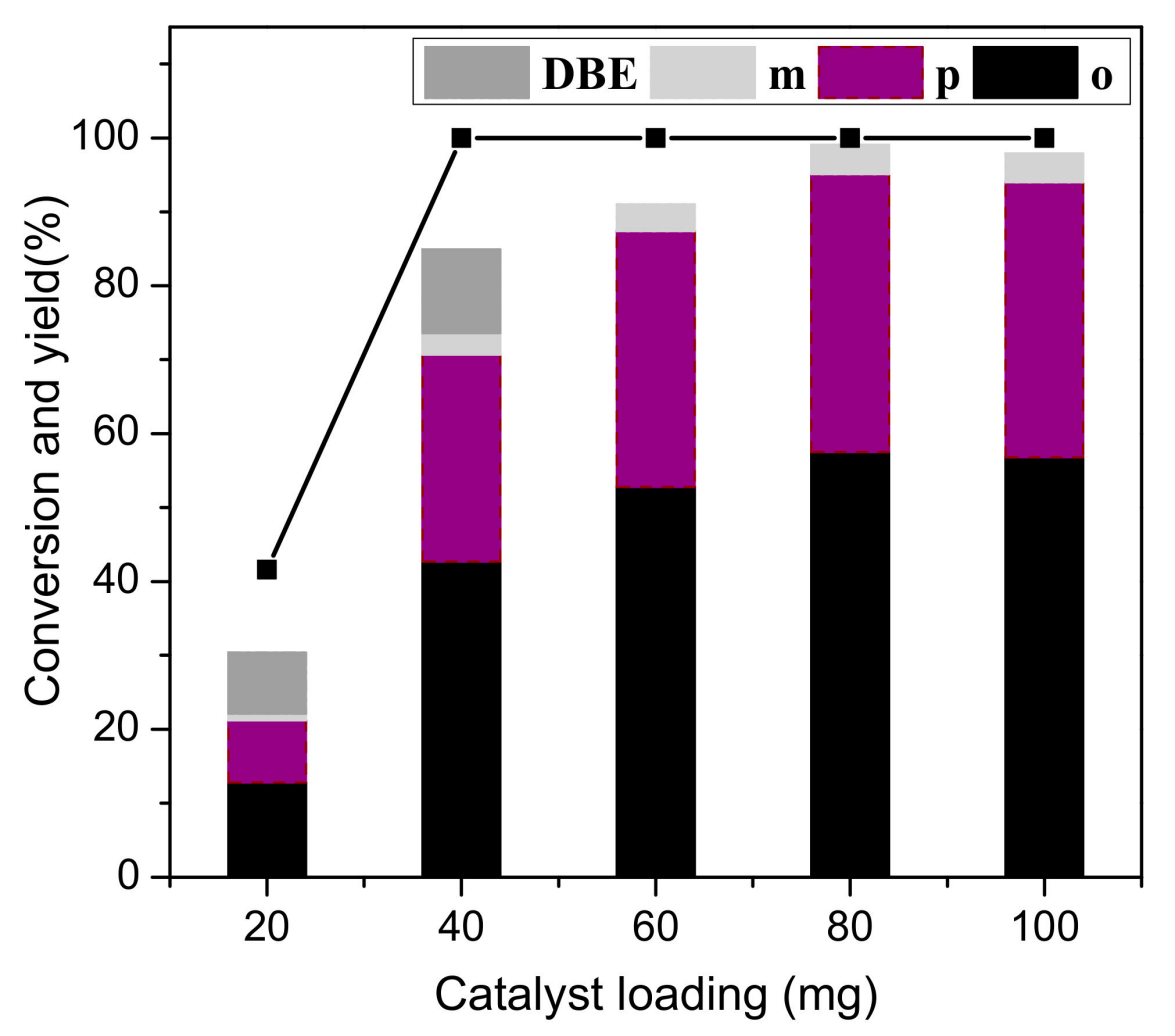
Influence of catalyst loading. Conditions: 0.5 mmol benzyl alcohol, 3 mL toluene, 120 °C, 6 h.

The catalyst loading affected the reaction rate due to the availability of active sites. Figure 6 shows the effect of the amount of catalyst on the catalytic performance over Hf/SBA-15(20). When the catalyst loading decreased to 20 mg (2.9 mol % Hf loading), limited benzyl alcohol conversion was obtained under the optimal reaction condition, together with a 22% yield of the targeted product. With gradual increase of the catalyst loading, the yield of benzylated products also increased, owing to more available acidic sites as the catalyst loading increased. A plateau was observed when the catalyst loading was 80 mg (11.5 mol % Hf), offering 99.1% of benzylated products. However, further increasing catalyst loading did not have much effect on the product yield and distributions. In consideration of the reaction efficiency and economy, 11.5 mol % Hf was selected as the optimal catalyst loading for other optimizations.

### 3.5. Effect of Reaction Temperature

**Figure 7:**
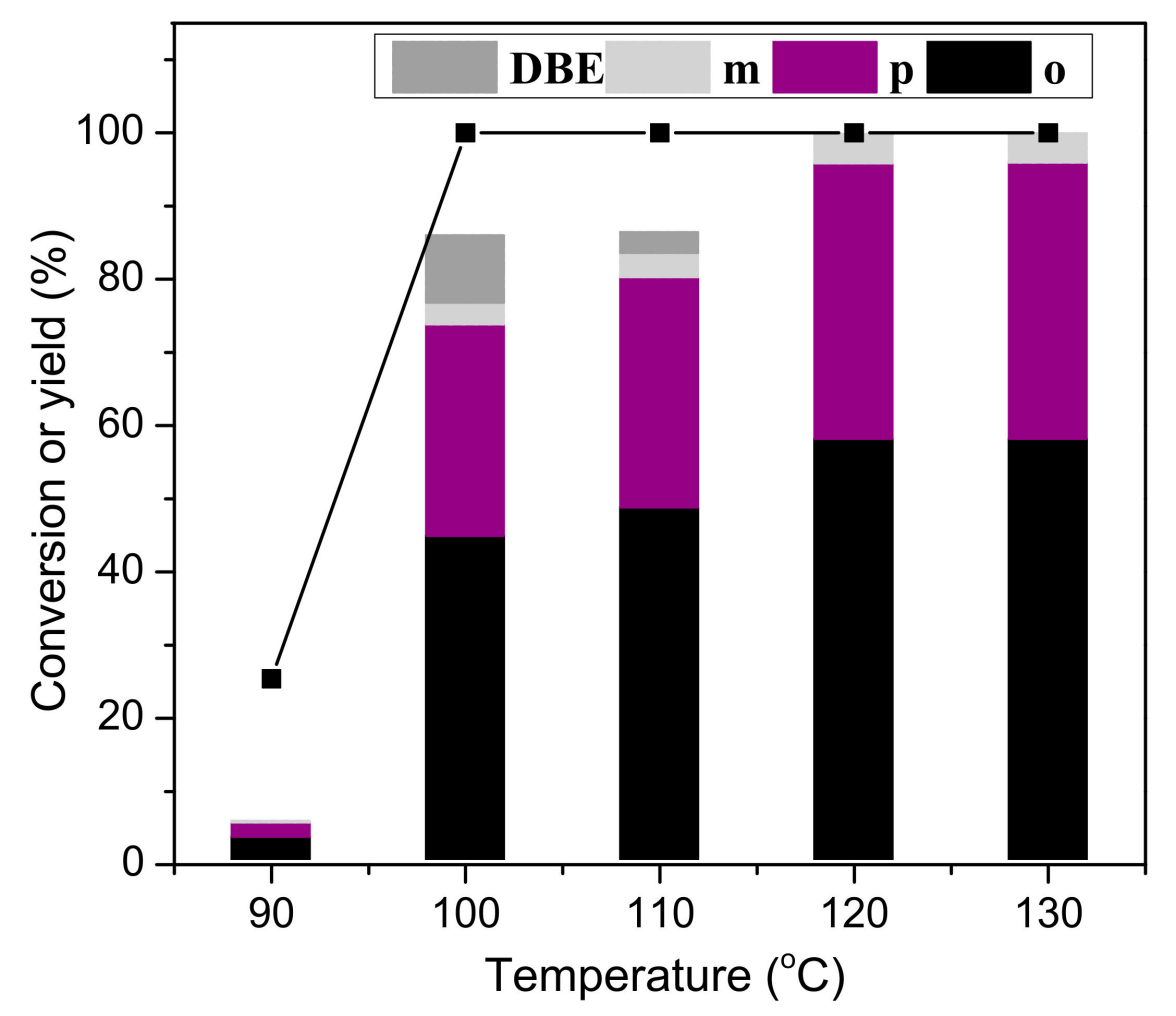
Influence of reaction temperature. Conditions: 0.5 mmol benzyl alcohol, 3 mL toluene, 6 h, 80 mg Hf/SBA-15(20).

The effect of temperature on the alkylation reaction was also evaluated, as shown in Figure 7. When the reaction temperature was conducted at a lower temperature of 90 °C, the conversion of benzyl alcohol was only 25.4%, and the yield of benzylated products was only 5.3%, without DBE formation. This result suggested a lower FC reaction rate at a lower reaction temperature, and the etherification reaction was suppressed at this temperature. When the temperature was slightly improved to 100 °C, the conversion of benzyl alcohol was remarkably enhanced to 100%, and the yield of the target product increased to 76.2%, together with 9.2% DBE. Further increasing the reaction temperature accelerated the FC reaction, while promoting the conversion of DBE with toluene, resulting in more target products. The highest yield of 99.1% could be obtained at 120 °C, and further increasing the temperature showed minor changes in product yields and distributions. Thus, the optimal reaction temperature was controlled to 120 °C.

### 3.6. Substrate Applications

After obtaining the optimal reaction condition, we also applied the catalytic system for the FC reaction with other substrates possessing different structures and functional groups (Table 3). For active primary benzyl alcohol, it showed high reactivity toward the FC reaction (Table 3, Entry 1). For a more specific substrate cinnamyl alcohol, the reaction can provide 63.1% of the alkylation products by prolonging the reaction time to 9 h (Table 3, Entry 2). Common aliphatic alcohols, such as cyclohexanol and 1-hexanol, were also tested, but no reaction occurred under the standard reaction condition (Table 3, Entries 3 and 4). This was probably attributed to the inert nature of these primary and secondary alcohols for the FC reaction, which were also mentioned in the reported literature [15]. In particular, alkyl halide benzyl bromide was also tested as an electrophile, which provided 99.1% of the target product under optimal reaction conditions, indicating the broad applicability of the Hf/SBA-15(20) catalyst in the FC reaction (Table 3, Entry 5). A series of aromatic substitutes, including ethylbenzene, mesitylene, m-xylene, and anisole, were also subjected to the reaction with benzyl alcohol, which also showed outstanding performances in the FC reaction, offering >96% product yields for all respective substrates, demonstrating the broad applicability of the Hf/SBA-15 catalyst in an FC reaction. However, for aromatics with electron-withdrawing functionalities, the reaction showed inferior results. It could only provide a 10.5% product yield when using bromobenzene as an electron-deficient aromatic compound for FC alkylation reaction. By increasing the reaction temperature to 160 °C, a 29% product yield could be obtained, which was also comparable to some of the previous work [5,12].

### 3.7. Catalyst Recycling

**Figure 8:**
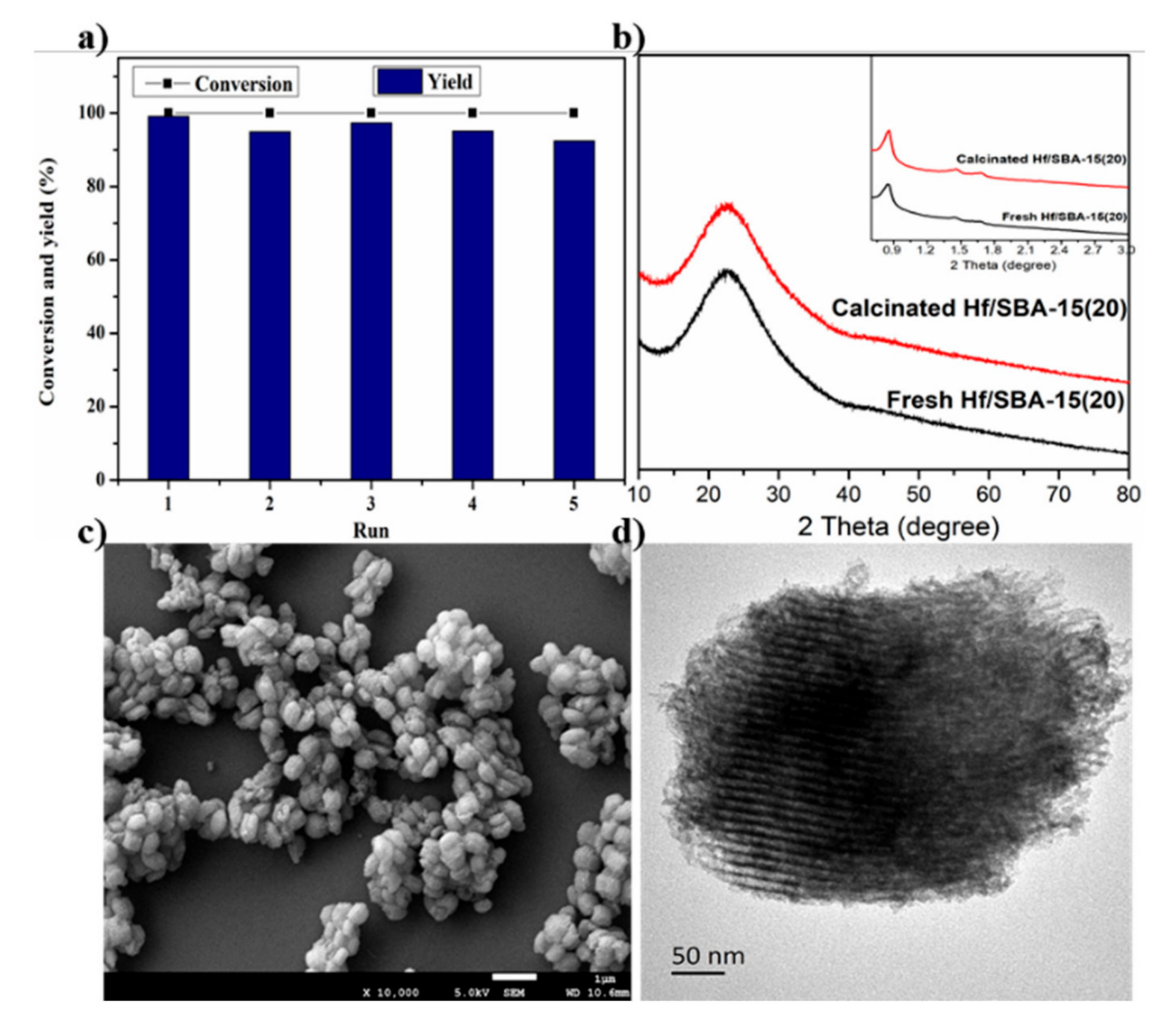
(**a**) Recycling of the calcinated Hf/SBA-15(20); (**b**) XRD characterization of the fresh and calcinated Hf/SBA-15(20); (**c**) SEM; (**d**) TEM.

The robustness and recyclability of the Hf/SBA-15(20) catalyst in the FC reaction was also investigated under the optimal reaction condition using a five-run recycling test. After each run, the solid catalyst was separated and calcined to completely remove organic residues on the catalyst surface to obtain the calcinated catalyst. Figure 8a presents the five-run test results, where the Hf/SBA-15(20) catalyst provided comparable product yields in each run. Slight decreases of the product yield in each run can be observed, which may be caused by the accumulated weight loss of the catalyst during the recycling process. To avoid the mask of catalyst deactivation due to excessive catalyst loading [23], the recycling test was also conducted with a lower catalyst loading of 60 mg (Figure S4), which showed a similar product variation trend to that from the optimal catalyst loading of 80 mg. This result clearly indicated that the optimal catalyst loading was appropriate to demonstrate the property and recycling ability of the catalyst. The calcinated catalyst was also subjected to SEM, TEM, and XRD characterizations to reveal structure changes during the recycling process. Very similar spectra were obtained as compared to the freshly prepared catalysts, suggesting the robustness of the Hf/SBA-15(20) catalyst.

**Figure 9:**
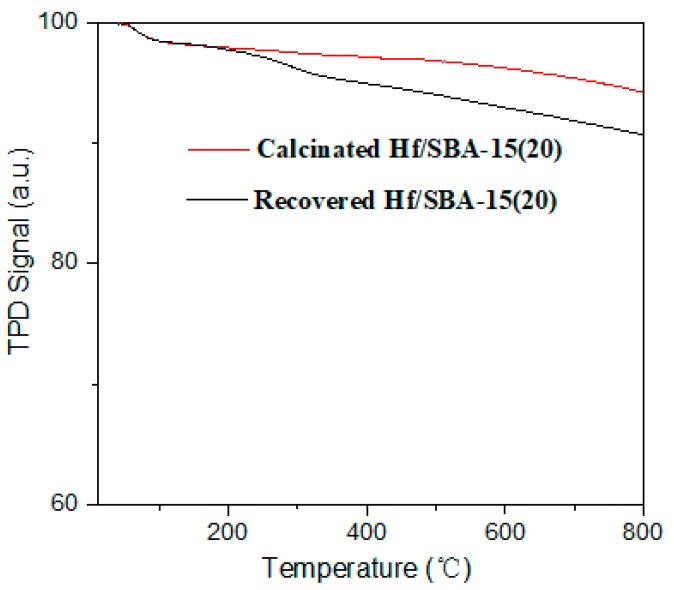
Thermogravimetric analysis (TGA) of the recovered and calcinated Hf/SBA-15(20) catalyst.

During the reaction, we also noticed that the recovered catalyst without calcination showed inferior catalytic activity, offering an 87% yield of product. This was probably caused by the deposition of the carbonaceous species and the absorption of formed water molecules that prevented mass diffusion and deactivated the acidic sites. To verify this, TGA characterizations of the recovered Hf/SBA-15(20) and calcinated Hf/SBA-15(20) were conducted to reveal their difference (Figure 9). A 9.4% weight loss was observed in the temperature ranging from 100 to 800 °C, for recovered Hf/SBA-15(20), whereas it was only 5.7% for the calcinated catalyst, indicating the organic carbonaceous species deposition on the catalyst surface. As for the influence of formed water, we carried out the experiments by adding a molecule sieve in the reaction system to remove the formed water (Figure S3). Accordingly, the model reaction with the molecule sieve showed a better reaction yield as compared to that without a molecule sieve at a comparatively low catalyst loading, which implied that the water did deactivate the acidic sites of the catalyst. However, this effect was minimized when the catalyst loading was increased. Thus, the deposition of carbonaceous species formed in the reaction would be a primary reason for the deactivation of the recovered catalyst. Future work would be dedicated to the exploration of a new supportive silica structure to create more robust and efficient catalysts.

## 4. Conclusions

In conclusion, we have developed a mesoporous Hf/SBA-15(20) catalyst for the FC alkylation reaction between benzyl alcohol and aromatic compounds, providing up to a 99.1% yield of diphenylmethane products under mild reaction conditions. Structure reactivity analysis showed that a mesoporous structure and well-dispersed Hf sites within a silica framework were crucial to the catalyst reactivity. The interaction between Hf sites and silica further led to the electron transfer from Hf metal center to silica, resulting in stronger Lewis acidity of Hf sites for the FC reaction. The catalyst also showed wide applicability in the FC alkylation reaction with several other substrates. Finally, Hf/SBA-15(20) provided good performances in the five-run recycling test, demonstrating the robust characteristic of the catalyst. This new Hf-doped mesoporous catalyst definitely adds to the catalyst catalogue for FC reactions, which may find more important applications in Lewis acid-catalyzed organic synthetic conversions in the future.

## Figures and Tables

**Table 1:** Textural properties of Hf/SBA-15 catalysts.

Catalysts	Surface BET (m^2^/g)	Pore Volume (cm^3^/g)	Pore Diameter (nm)
Hf/SBA-15(15)	624.4	1.1	10.3
Hf/SBA-15(20)	724.2	1.3	10.4
Hf/SBA-15(25)	774.7	1.3	10.3
Hf/SBA-15(30)	811.3	1.4	10.0

**Table 2:** Alkylation reactions over different solid acid catalysts ^a^.

Entry	Catalyst	Conversion (%)	BA Yield (%)	DBE Yield (%)	o/p/m
1	SBA-15	0	0	-	-
2 ^b^	Zr/SBA-15(20)	100	89.3	-	57:39:4
3 ^b^	W/SBA-15(20)	100	83.9	-	55:40:5
4 ^b^	Fe/SBA-15(20)	0	0	-	-
5	Hf/SBA-15(15)	100	92.4	-	57:39:4
6 ^c^	Hf/SBA-15(20)	100	99.1 (98.0)	-	58:38:4
7	Hf/SBA-15(25)	100	94.2	-	57:38:5
8	Hf/SBA-15(30)	100	94.7	-	57:38:5
9	HfCl_4_	0	0	-	-
10	HfO_2_	0	0	-	-
11	HfO(PO_4_)_2_	86.1	2.7	5.2	56:40:4
12	HfO(OH)_2_	0	0	-	-
13	Amberlyst-15	100	59	-	55:40:5
14	HZSM-5	58.8	11.3	13.7	56:39:5
15	H-β	100	77.7	-	57:39:4

^a^: Reaction conditions: 0.5 mmol benzyl alcohol, 3 mL toluene, 11.5 mol % metal loading, 120 °C, 6 h. GC yields. ^b^: Si/metal ratio = 20:1. ^c^: Isolated yield in parentheses.

**Table 3:** Catalytic activity of Hf/SBA-15(20) on the Friedel–Crafts alkylation reactions between different alcohols and aromatics ^a^.

Entry	Alcohol	Aromatics	Alcohol Conversion (%)	GC Yield (%)	Isolated Yield (%)	o/p/m ^c^
1	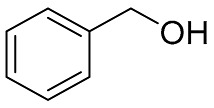	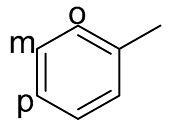	100	99.1	98.0	58:38:4
2 ^b^	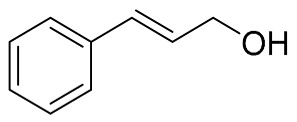	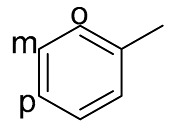	100	65.8	63.1	76:22:1
3	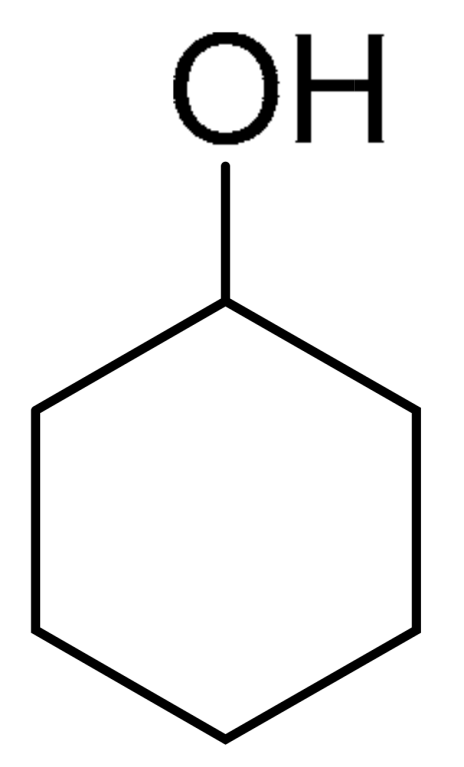	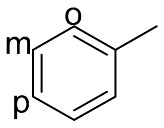	0	0	-	-
4	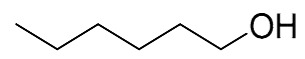	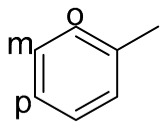	0	0	-	-
5	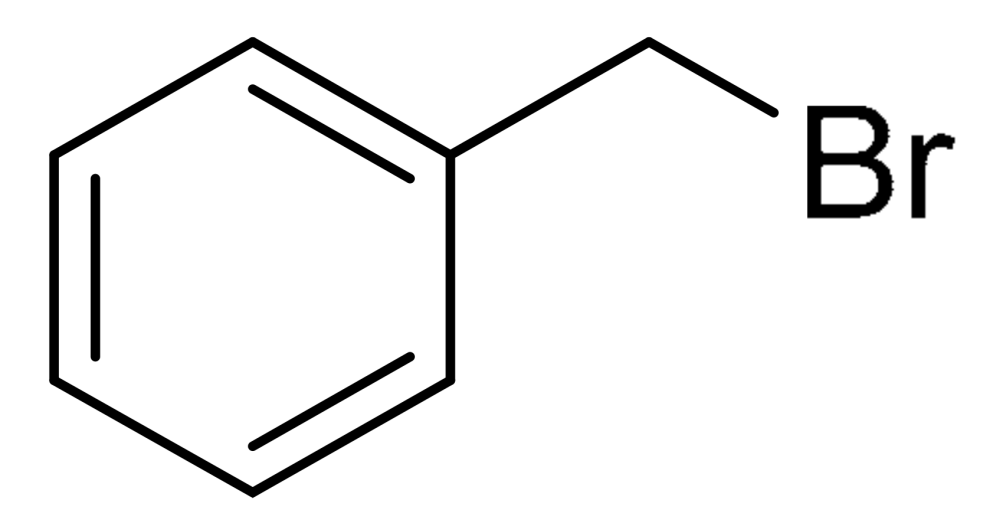	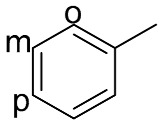	100	99.5	99.1	58:38:4
6	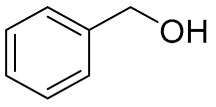	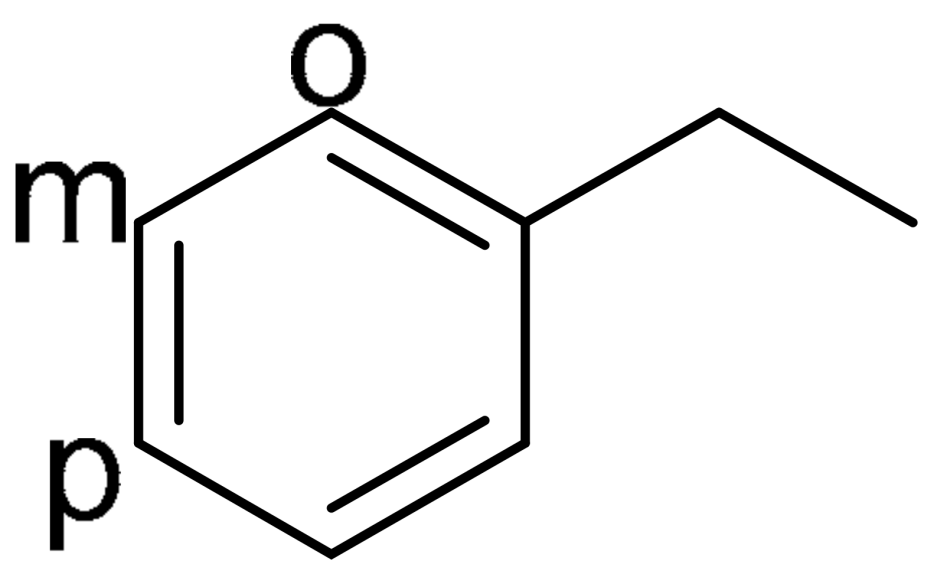	100	99.0	98.2	61:36:3
7	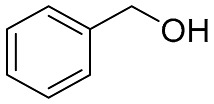	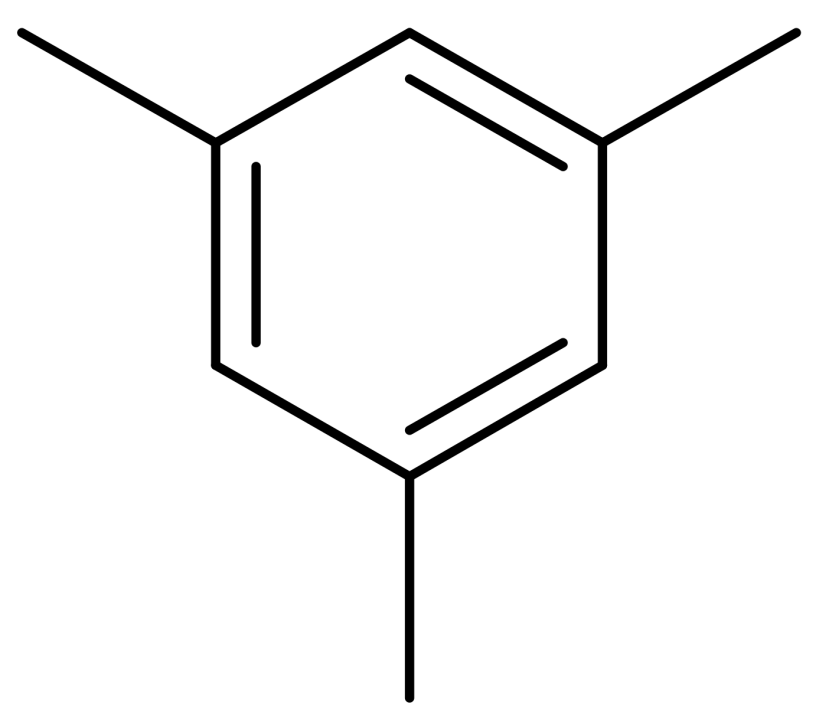	100	99.0	97.5	-
8	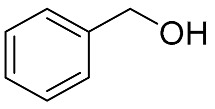	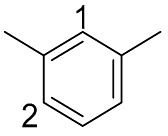	100	96.6	95.1	20:80 (1:2)
9	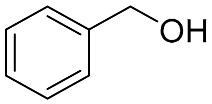	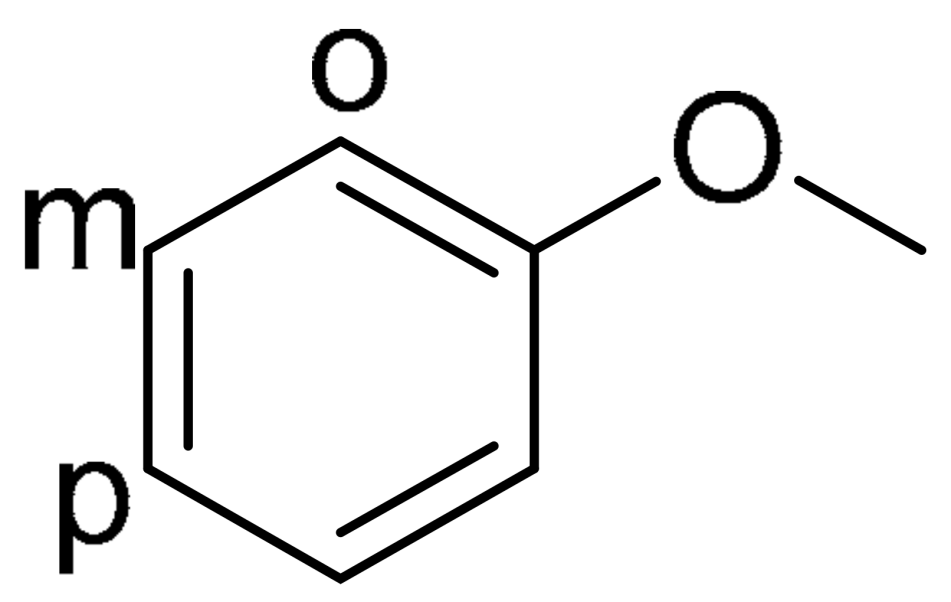	100	98.1	97.5	55:45:0
10 ^b^	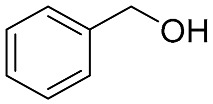	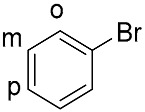	100	11.7 (31.2)	10.5 (29.0)	52:48:0

^a^: Reaction condition: 0.5 mmol alcohols, 3 mL aromatics, 120 °C, 6 h, 80 mg Hf/SBA-15(20); ^b^: 9h; ^c^: Isomer ratios were determined by ^1^H-NMR; ^b^: 160 °C reaction temperature was used in the parentheses.

## Supplementary Materials

The following are available online at https://www.mdpi.com/2079-4991/9/8/1128/s1, Figure S1: Reaction evolution over a) Zr/SBA-15 catalyst and b) Hf/SBA-15, Figure S2: Pyridine-IR characterization of Hf/SBA-15 and Zr/SBA-15 catalysts, Figure S3: The influence of molecular sieve on reaction, Figure S4: Catalyst recycling test with 60mg loading of Hf/SBA-15 catalyst under optimized condition, Table S1: FC benzylation of aromatics over different catalysts, Table S2: Reaction evolution over Hf/SBA-15(20) catalyst at different time, Table S3: Reaction evolution over Zr/SBA-15(20) catalyst at different time, Table S4: Influence of catalyst loading on the reaction over Hf/SBA-15(20), Table S5: Influence of reaction temperature on the reaction over Hf/SBA-15(20), Table S6: Influence of aromatic loading on the reaction efficiency, Scheme S1: Catalytic condensation of furfural with 2-methylfuran over Hf/SBA-15(20) and Zr/SBA-15(20) catalyst, NMR spectra of the benzylated products.
