# Supplementary material for: Hafnium-Doped Mesoporous Silica as Efficient Lewis Acidic Catalyst for Friedel–Crafts Alkylation Reactions
Source: Nanomaterials (Basel). 2019 Aug 5;9(8):1128. doi: 10.3390/nano9081128 (PMC6723505; doi:10.3390/nano9081128)
Supplement: Supplementary file 1 [file nanomaterials-09-01128-s001.pdf]

Supporting Information

Hafnium Doped Mesoporous Silica as Efficient Lewis Acidic Catalyst for Friedel-Crafts Alkylation Reactions

Yao-Bing Huang [1,2,3,*], Yu-Jia Luo [2], Fei Wang [1,*]

[1] Jiangsu Co-Innovation Center of Efficient Processing and Utilization of Forest Resources, Nanjing Forestry University, Nanjing 210037, China

[2] College of Chemical Engineering, Nanjing Forestry University, Nanjing 210037, China

[3] Department of Chemical and Biomolecular Engineering, University of California-Berkeley, Berkeley 94706, California, USA

1. Table S1. FC benzylation of aromatics over different catalysts.
2. Table S2. Reaction evolution over Hf/SBA-15(20) catalyst at different time.
3. Table S3. Reaction evolution over Zr/SBA-15(20) catalyst at different time.
4. Table S4. Influence of catalyst loading on the reaction over Hf/SBA-15(20).
5. Table S5. Influence of reaction temperature on the reaction over Hf/SBA-15(20).
6. Table S6. Influence of aromatic loading on the reaction efficiency.
7. Scheme S1. Catalytic condensation of furfural with 2-methylfuran over Hf/SBA-15(20) and Zr/SBA-15(20) catalyst.
8. Figure S1. Reaction evolution over a) Zr/SBA-15 catalyst and b) Hf/SBA-15.
9. Figure S2. Pyridine-IR characterization of Hf/SBA-15 and Zr/SBA-15 catalysts.
10. Figure S3. The influence of molecular sieve on reaction.
11. Figure S4. Catalyst recycling test with 60mg loading of Hf/SBA-15 catalyst under optimized condition.
12. NMR spectra of the benzylated products.

Table S1. FC benzylation of aromatics over different catalysts.

**Catalyst**	**Acid type (B or L)**	**Temp. (°C)**	**Time (h)**	**BA yield (%)**	**o:p:m**	**Ref.**
Al/SBA-15	L	110	24	>95	52:48:0	[1]
Triphenylphosphine ditriflate	B	25	0.5	84	42:58:0	[2]
M-ZrO_2 /SO_4^{2-}	B	150	2	77	37:40:0	[3]
MoO_3/M-ZrPO	B-L	110	2	45.9	33:28:0	[4]
$Hf_{0.5}$[TEAPS]$PW_{12}O_{40}$	B-L	140	2	91.7	47:53:0	Ref 5
TPA–PANI	B	80	4	98	-	Ref 9
Au/SiO_2 IL	L	65	2	77.5	-	Ref 12
Sn_1TPA	B	120	1	99	-	Ref 13
$Sc(OTf)_3$(25)/MCM-41	B	80	1.5	95	1:1:0	Ref 14
SiO_2–ZrO_2	L	110	15	32	-	Ref 15
Fe-TUD-1	B	110	1	90	-	Ref 21
Hf/SBA-15(20)	L	120	6	99.1		This work

Table S2. Reaction evolution over Hf/SBA-15(20) catalyst at different time.

**Entry**	**Time (h)**	**Conv (%)**	**BA yield(%)**	**o:p:m**	**DBE yield(%)**
1	1	36.3	19.9	11.5:7.6:0.8	5.9
2	2	66.9	47.5	27.6:18:1.9	10.4
3	3	100	86.5	50.2:32.9:3.5	3.6
4	4	100	90.3	52.4:34.3:3.6	/
5	5	100	93.3	54.1:35.5:3.7	/
6	6	100	99.1	57.5:37.6:4	/

Reaction conditions: 0.5 mmol benzyl alcohol, 3 mL toluene, 80 mg Hf/SBA-15(20), 11.5% Zr loading, 120 °C.

Table S3. Reaction evolution over Zr/SBA-15(20) catalyst at different time.

**Entry**	**Time (h)**	**Conv (%)**	**BA yield (%)**	**o:p:m**	**DBE yield (%)**
1	1	28.3	8.9	5.2:3.4:0.4	4.8
2	2	38.7	17.1	9.9:6.5:0.7	7.9
3	3	60.2	44	25.5:16.7:1.8	10
4	4	88.1	73.9	42.9:28.1:3	3.1
5	5	93.5	80.2	46.5:30.5:3.2	-
6	6	100	89.3	51.8:33.9:3.6	-

Reaction conditions: 0.5 mmol benzyl alcohol, 3 ml toluene, 11.5 mol% Zr loading, 120 °C.

Table S4. Influence of catalyst loading on the reaction over Hf/SBA-15(20).

Entry	Catalyst amount (mg)	Conv (%)	BA yield (%)	o:p:m	DBE yield (%)
1	20	41.6	22	12.8:8.4:0.9	8.3
2	40	100	73.6	42.7:28:2.9	11.3
3	60	100	91.1	52.8:34.6:3.6	/
4	80	100	99.1	57.5:37.6:4	/
5	100	100	97.9	56.8:37.2:3.9	/

Reaction conditions: 0.5 mmol benzyl alcohol, 3 mL toluene, 6 h， 120 ºC.

Table S5. Influence of reaction temperature on the reaction over Hf/SBA-15(20).

Entry	Temperature (ºC)	Conv (%)	BA yield(%)	o:p:m	DBE yield(%)
1	90	25.4	5.3	3.1:2:0.2	/
2	100	100	76.2	44.2:28.9:3	9.2
3	110	100	83	48.1:31.5:3.3	2.8
4	120	100	99.1	57.5:37.6:4	/
5	130	100	99.2	57.5:37.7:4	/

Reaction conditions: 0.5 mmol benzyl alcohol, 3 mL toluene, 80 mg Hf/SBA-15(20)， 6 h.

Table S6. Influence of aromatic loading on the reaction efficiency.

Entry	toluene (mL)	Time (h)	Conv (%)	BA yield (%)	DBE yield (%)
1	1	6	100	63.9	-
2	1	14	100	83.9	-
3	1.5	6	100	78.6	-
4	1.5	14	100	91.6	-

Reaction conditions: 0.5 mmol benzyl alcohol, 80 mg Hf/SBA-15(20), 120 °C.

Catalytic test

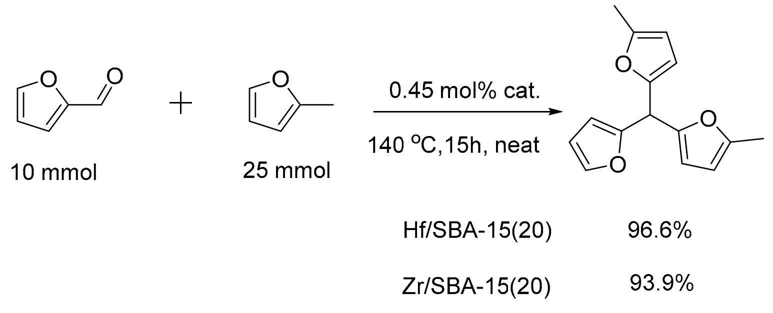

Scheme S1. Catalytic condensation of furfural with 2-methylfuran over Hf/SBA-15(20) and Zr/SBA-15(20) catalyst.

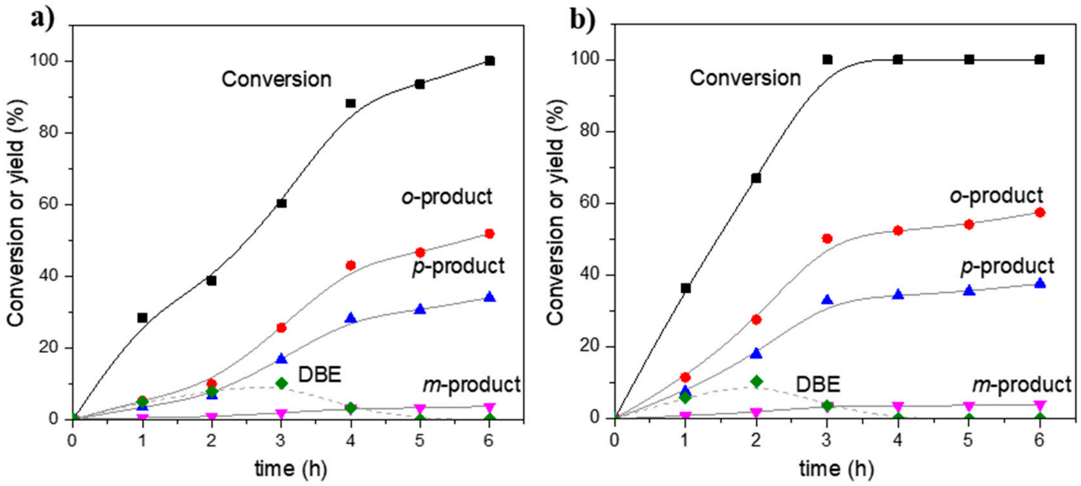

Figure S1. Reaction evolution over a) Zr/SBA-15 catalyst and b) Hf/SBA-15. Conditions: 0.5 mmol benzyl alcohol, 3 ml toluene, 120 °C, 11.5 mol% metal loading.

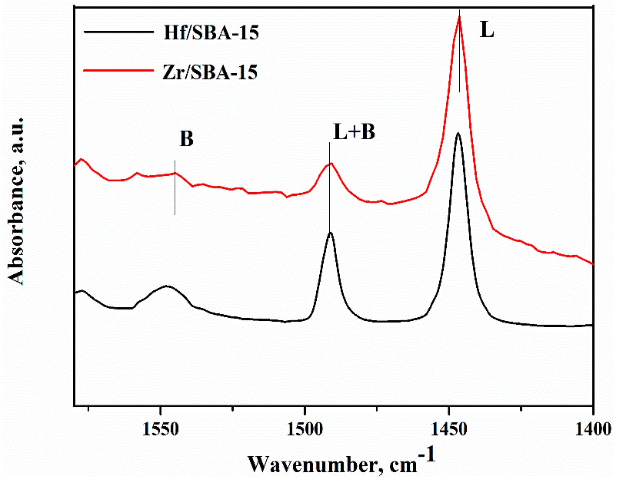

catalyst	B acidity (mmol/g)	L+B acidity (mmol/g)	L acidity (mmol/g)	Total acidity (mmol/g)
Hf/SBA-15	4.3	4.5	8.7	17.5
Zr/SBA-15	2.4	3.9	7.7	14.0

Figure S2. Pyridine-IR characterization of Hf/SBA-15 and Zr/SBA-15 catalysts.

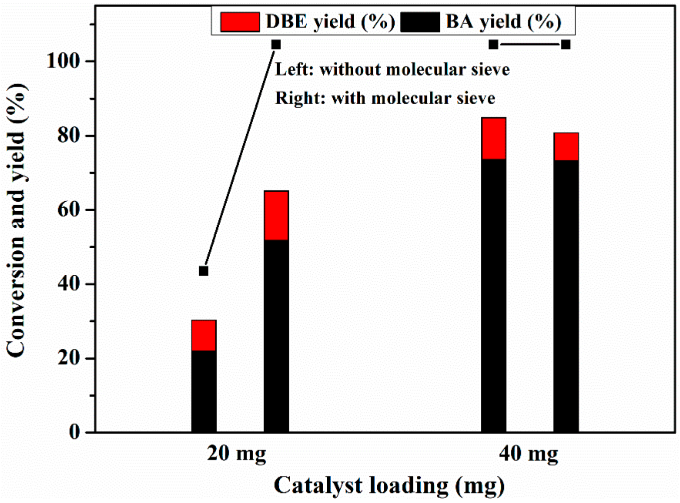

Figure S3. The influence of molecular sieve on reaction. Reaction conditions: 0.5 mmol benzyl alcohol, 3 ml toluene, Hf/SBA-15 80 mg, 4A molecular sieve 100 mg, 120 °C, 6 h.

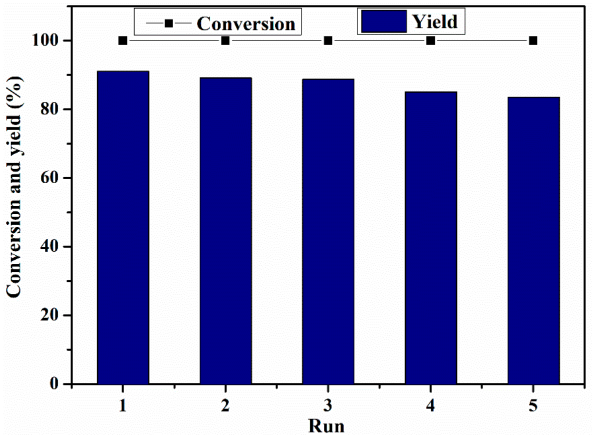

Figure S4. Catalyst recycling test with 60mg loading of Hf/SBA-15 catalyst under optimized condition. Reaction conditions: 0.5 mmol benzyl alcohol, 3 ml toluene, Hf/SBA-15 60 mg, 120 °C, 6 h.

Reference

[1] Gracia, M.J.; Losada, E.; Luque, R.; Campelo, J.M.; Luna, D.; Marinas, J.M.; Romero, A.A. Activity of Gallium and Aluminum SBA-15 materials in the Friedel–Crafts alkylation of toluene with benzyl chloride and benzyl alcohol. Applied Catalysis A: General, 2008, 349, 148–155.

[2] Khodaei, M.M.; Nazari, E. Synthesis of diarylmethanes via a Friedel–Crafts benzylation using arenes and benzyl alcohols in the presence of triphenylphosphine ditriflate. Tetrahedron Letters, 2012, 53, 5131–5135.

[3] Miao, Z.; Zhou, J.; Zhao, J.; Liu, D.; Bi, X.; Chou, L.; Zhuo, S. A novel mesoporous sulfated zirconium solid acid catalyst for Friedel-Crafts benzylation reaction. Applied Surface Science, 2017, 411, 419–430.

[4] Miao, Z.; Li, Z.; Zhao, J.; Si, W.; Zhou, J.; Zhuo, S. MoO3 supported on ordered mesoporous zirconium oxophosphate: An efficient and reusability solid acid catalyst for alkylation and esterification. Molecular Catalysis, 2018, 444, 10–21.

NMR spectra of the benzylated products

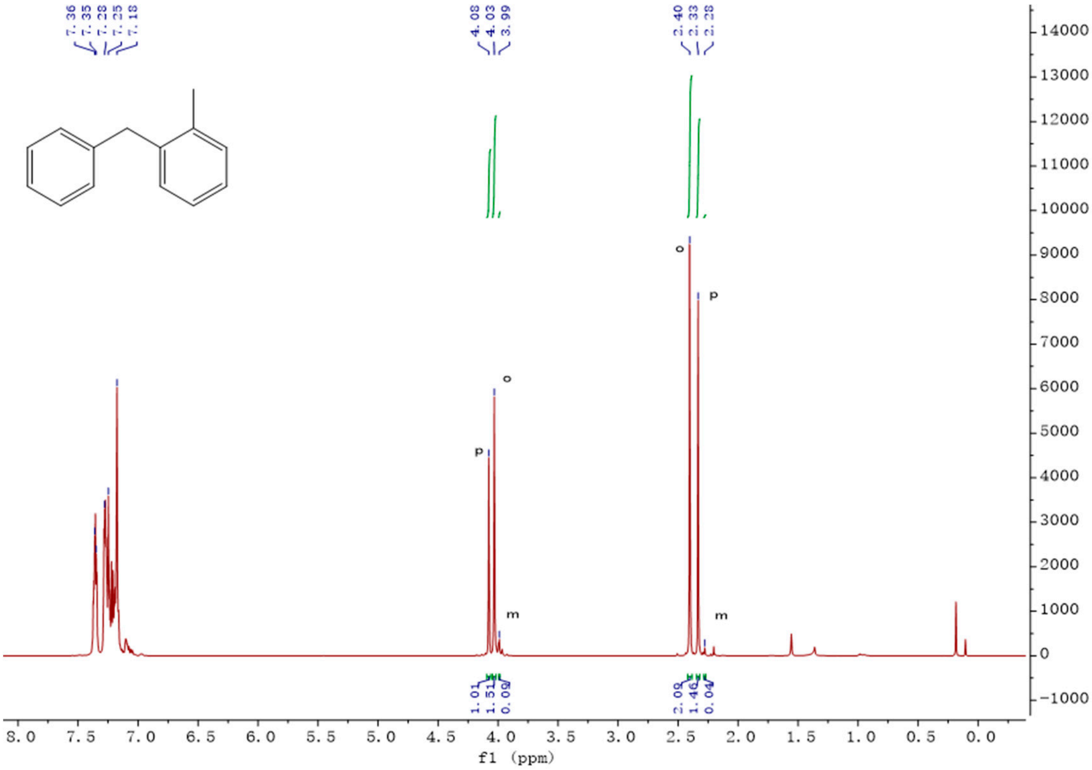

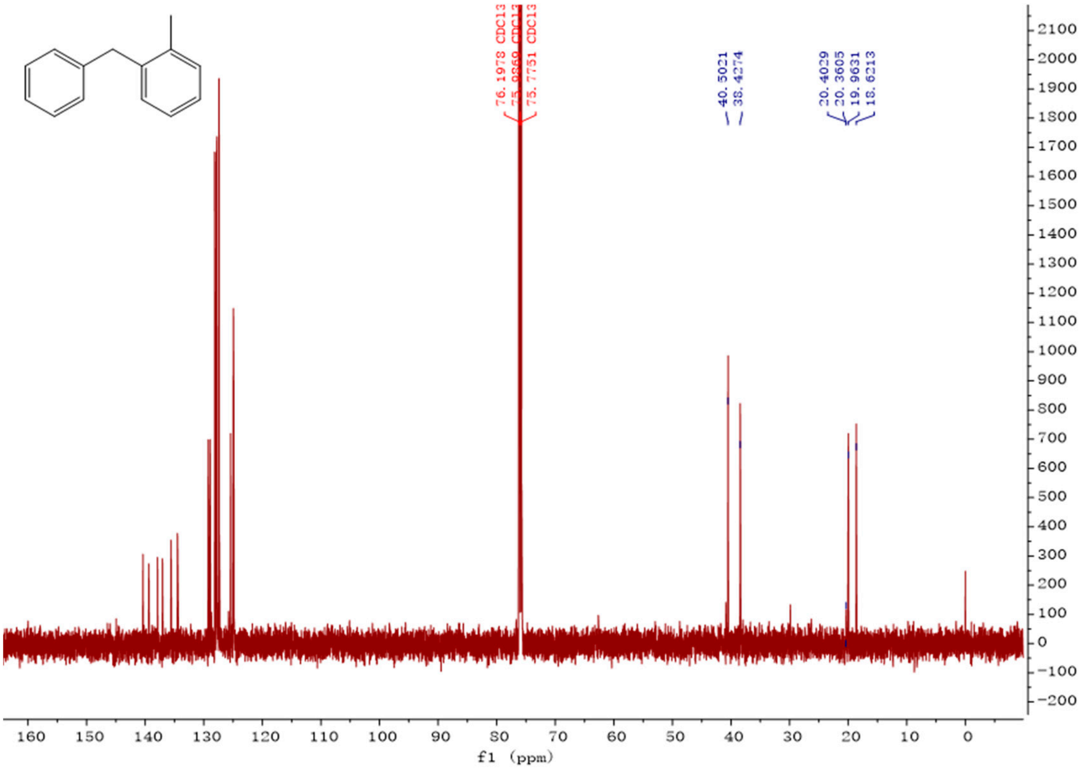

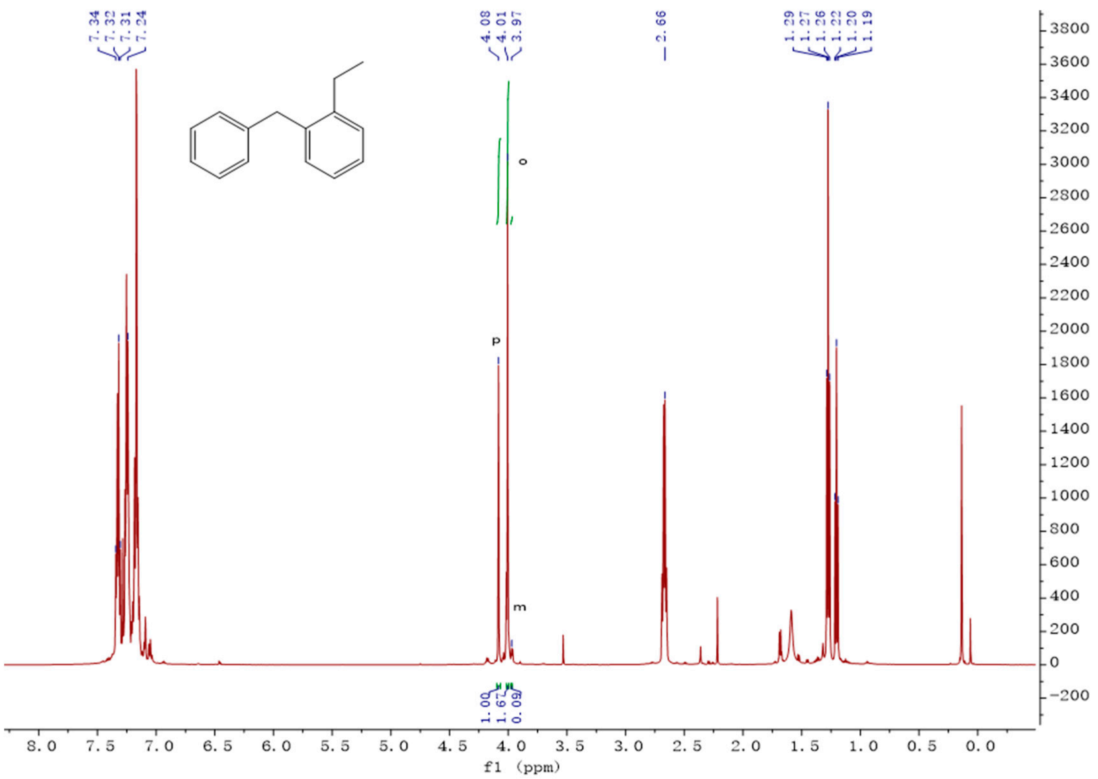
7.34
7.32
7.31
7.24
4.08
4.01
3.97
2.66
1.29
1.27
1.26
1.22
1.20
1.19
o
p
m
1.00
1.67
0.09
f1 (ppm)

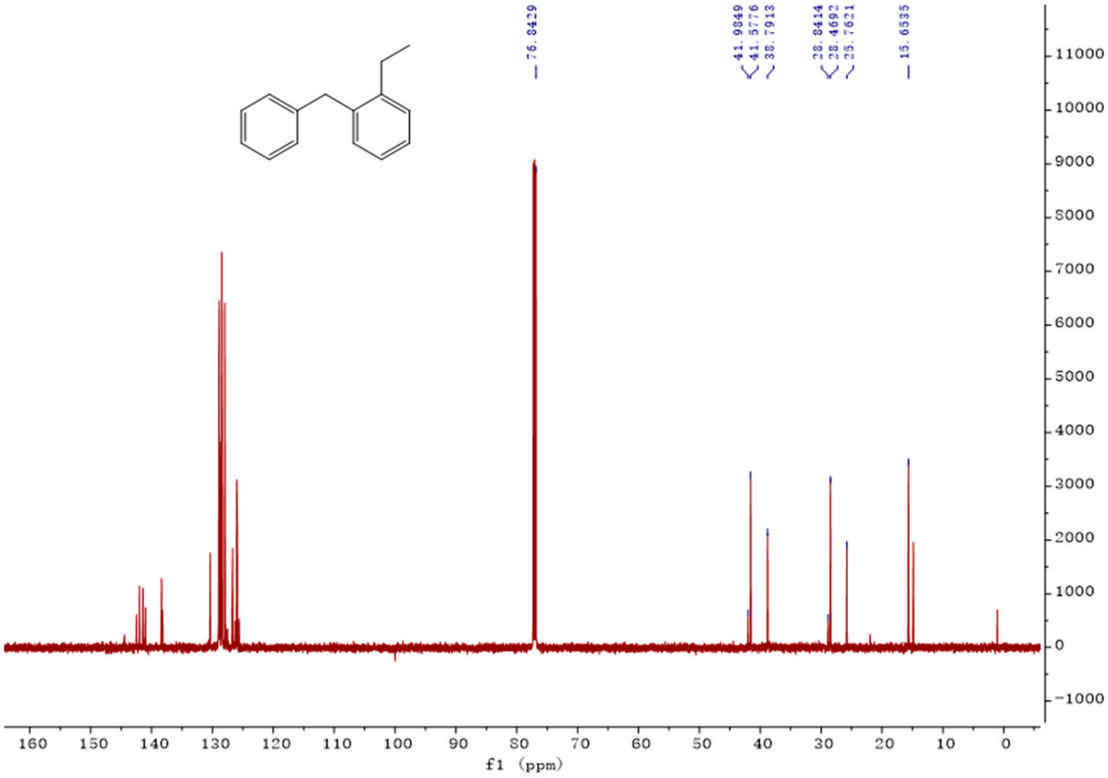
76.8429
41.9849
41.5776
38.7913
28.8414
28.4692
25.7621
15.6535
f1 (ppm)

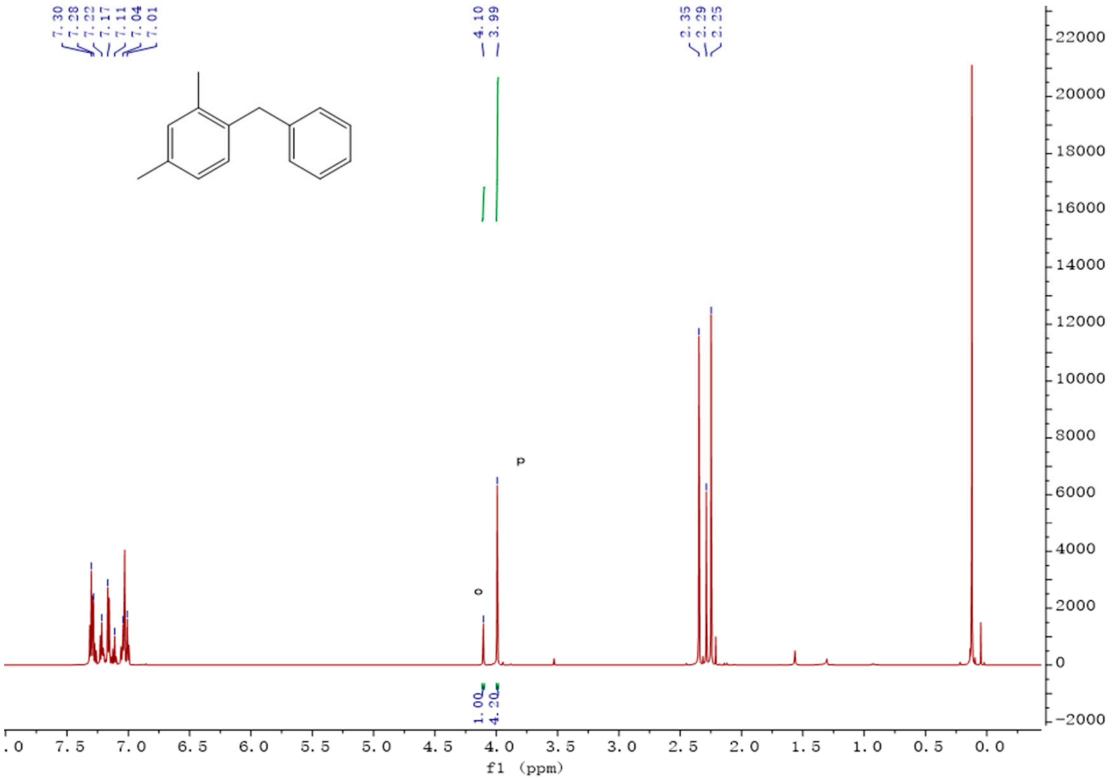
7.30
7.28
7.22
7.17
7.11
7.04
7.01
4.10
3.99
2.35
2.29
2.25
o
p
1.00
4.20
f1 (ppm)

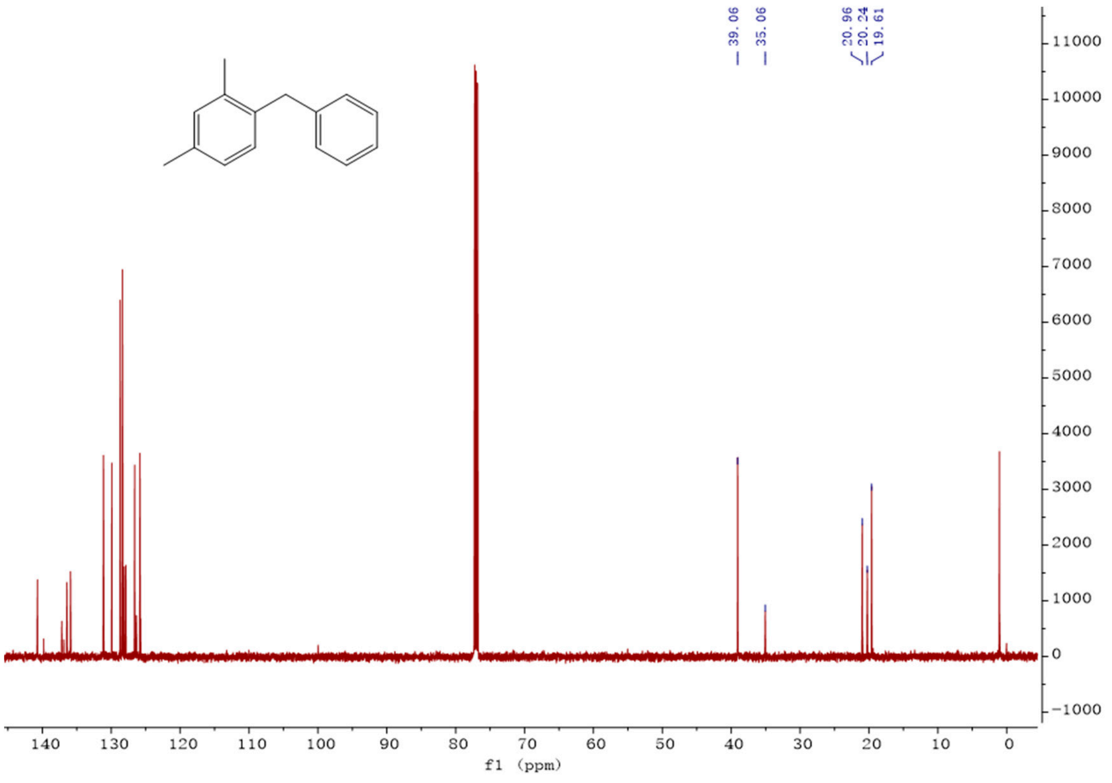
39.06
35.06
20.96
20.24
19.61
f1 (ppm)

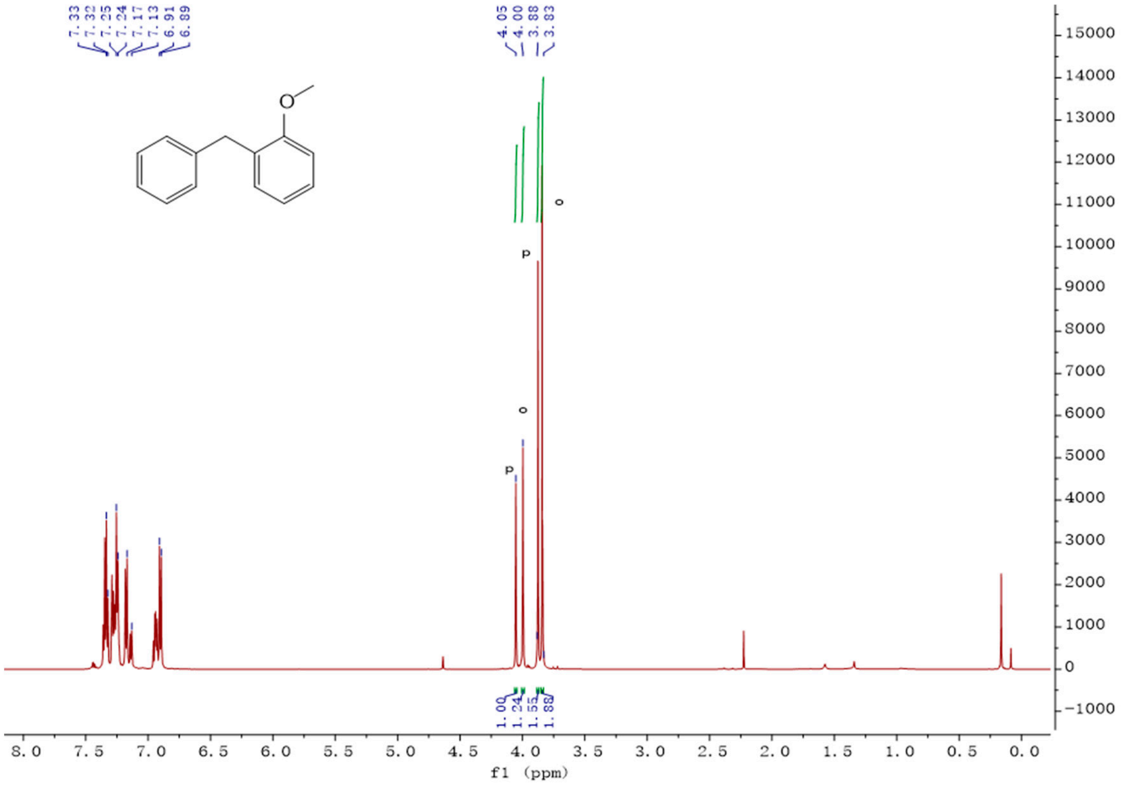

7.33
7.32
7.25
7.24
7.17
7.13
6.91
6.89
4.05
4.00
3.88
3.83
1.00
1.24
1.55
1.88
f1 (ppm)

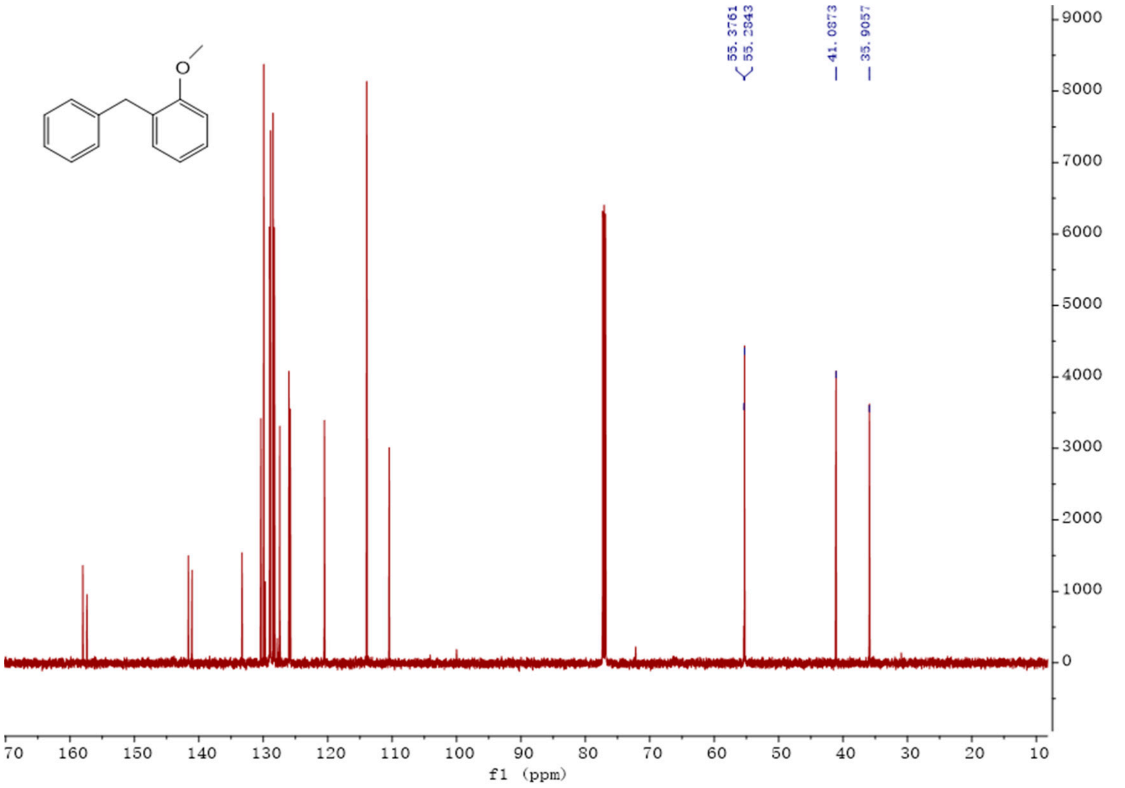

55.3761
55.2843
41.0873
35.9057
f1 (ppm)

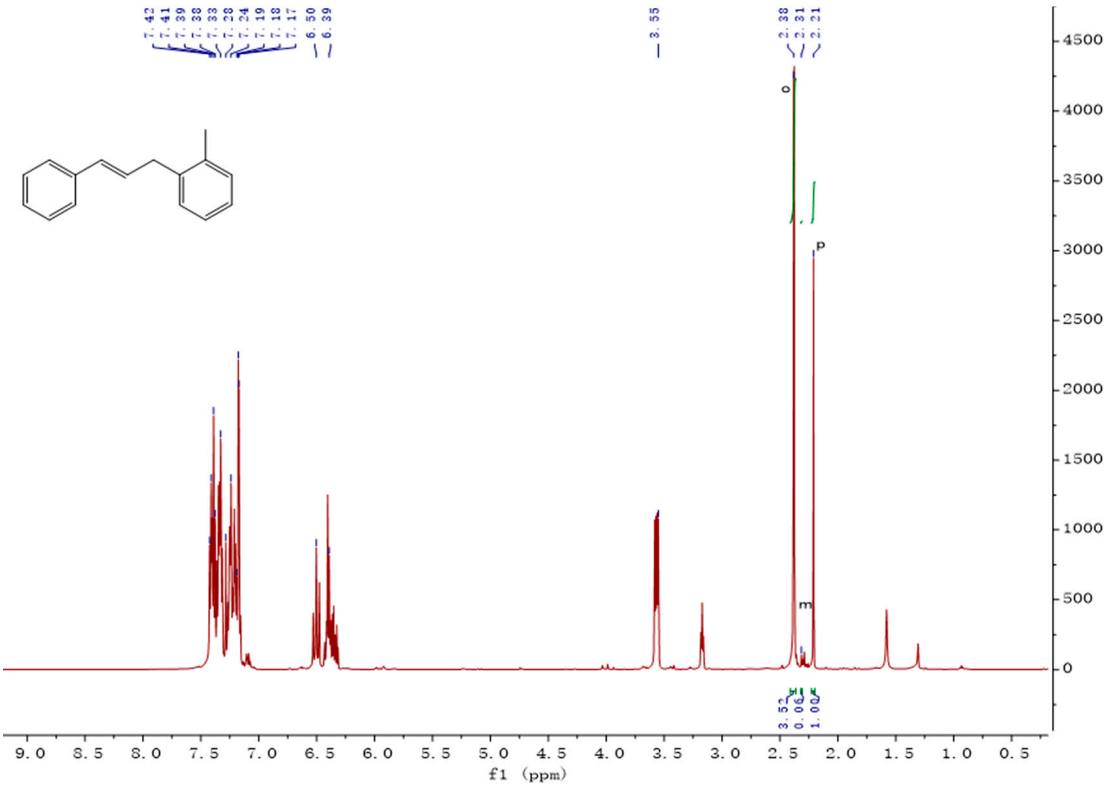
7.42
7.41
7.39
7.38
7.33
7.28
7.24
7.19
7.18
7.17
6.50
6.39
3.55
2.38
2.31
2.21
o
p
m
3.52
0.06
1.00
4500
4000
3500
3000
2500
2000
1500
1000
500
0
9.0 8.5 8.0 7.5 7.0 6.5 6.0 5.5 5.0 4.5 4.0 3.5 3.0 2.5 2.0 1.5 1.0 0.5
f1 (ppm)

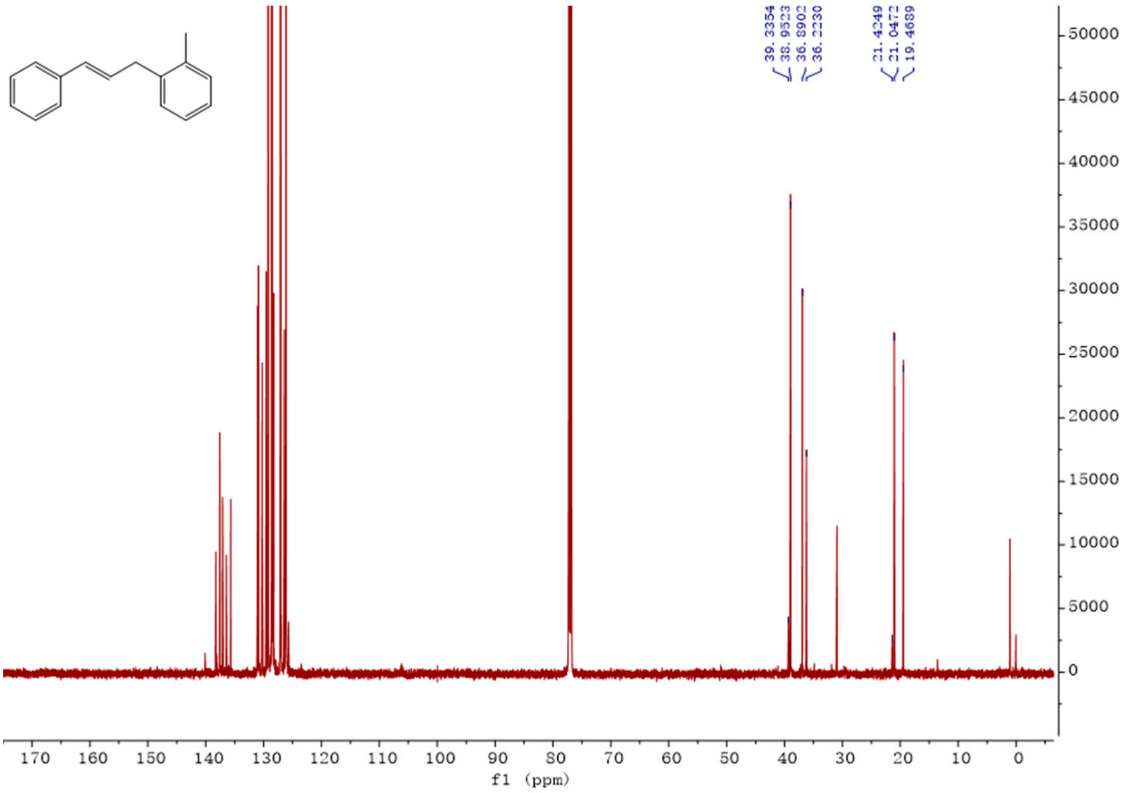
39.3354
38.9523
36.8902
36.2230
21.4249
21.0472
19.4689
50000
45000
40000
35000
30000
25000
20000
15000
10000
5000
0
170 160 150 140 130 120 110 100 90 80 70 60 50 40 30 20 10 0
f1 (ppm)

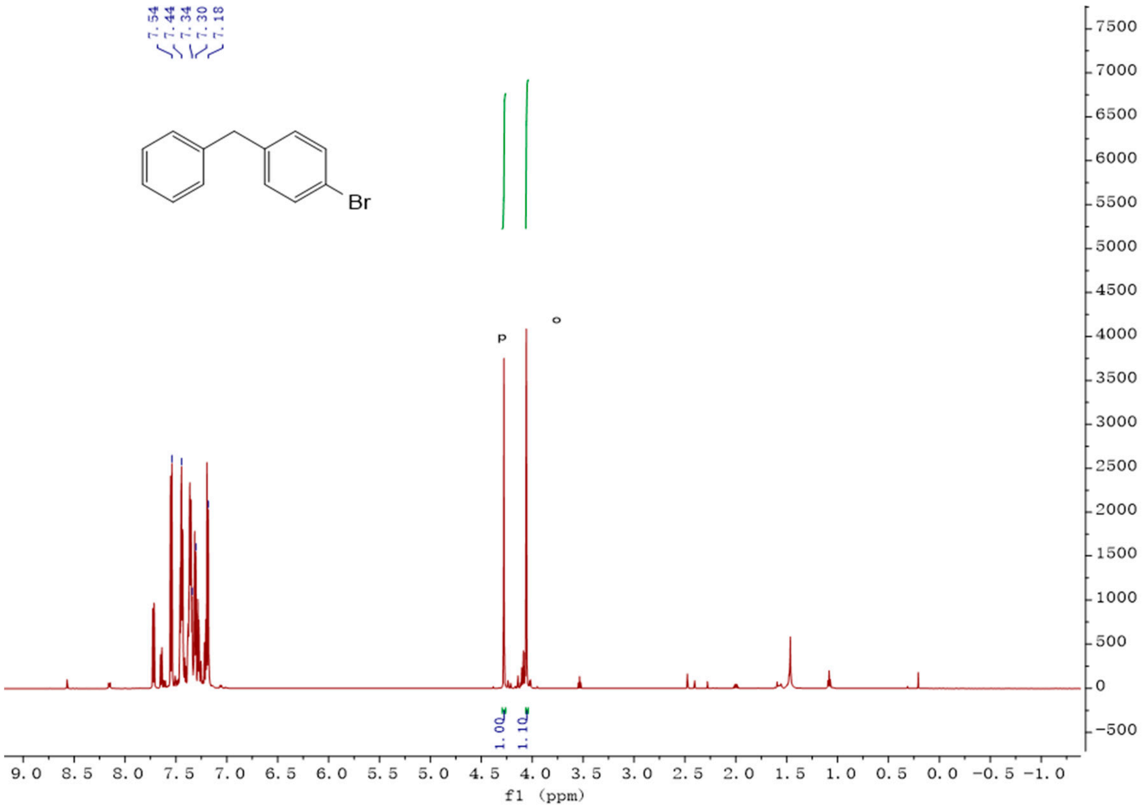
7.54
7.44
7.34
7.30
7.18
Br
p
o
1.00
1.10
f1 (ppm)

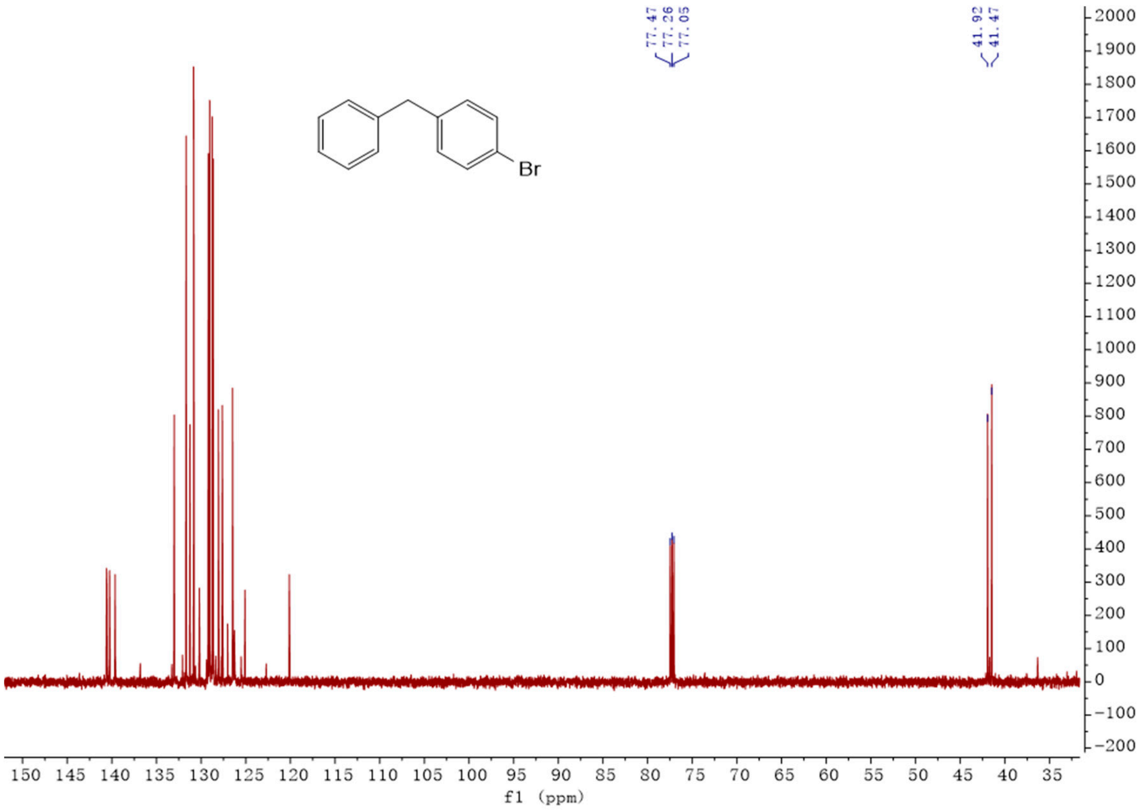
77.47
77.26
77.05
41.92
41.47
Br
f1 (ppm)
